# IκBα is required for full transcriptional induction of some NFκB-regulated genes in response to TNF in MCF-7 cells

**DOI:** 10.1038/s41540-021-00204-7

**Published:** 2021-12-01

**Authors:** Minami Ando, Shigeyuki Magi, Masahide Seki, Yutaka Suzuki, Takeya Kasukawa, Diane Lefaudeux, Alexander Hoffmann, Mariko Okada

**Affiliations:** 1 Institute for Protein Research, Osaka University, 3-2 Yamadaoka, Suita, Osaka 565-0871 Japan; 2 Department of Computational Biology and Medical Sciences, Graduate School of Frontier Sciences, The University of Tokyo, Kashiwa, Chiba 277-8568 Japan; 3 RIKEN Center for Integrative Medical Sciences, Yokohama, Kanagawa 230-0045 Japan; 4 UCLA Department of Microbiology, Immunology and Molecular Genetics, 570 Boyer Hall, Los Angeles, CA 90095 USA; 5 Center for Drug Design and Research, National Institutes of Biomedical Innovation, Health and Nutrition, Ibaraki, Osaka 567-0085 Japan; 6 Institute for Chemical Research, Kyoto University, Kyoto, 611-0011 Japan

**Keywords:** Regulatory networks, Molecular biology

## Abstract

Inflammatory stimuli triggers the degradation of three inhibitory κB (IκB) proteins, allowing for nuclear translocation of nuclear factor-κB (NFκB) for transcriptional induction of its target genes. Of these three, IκBα is a well-known negative feedback regulator that limits the duration of NFκB activity. We sought to determine whether IκBα’s role in enabling or limiting NFκB activation is important for tumor necrosis factor (TNF)-induced gene expression in human breast cancer cells (MCF-7). Contrary to our expectations, many more TNF-response genes showed reduced induction than enhanced induction in IκBα knockdown cells. Mathematical modeling was used to investigate the underlying mechanism. We found that the reduced activation of some NFκB target genes in IκBα-deficient cells could be explained by the incoherent feedforward loop (IFFL) model. In addition, for a subset of genes, prolonged NFκB activity due to loss of negative feedback control did not prolong their transient activation; this implied a multi-state transcription cycle control of gene induction. Genes encoding key inflammation-related transcription factors, such as *JUNB* and *KLF10*, were found to be best represented by a model that contained both the IFFL and the transcription cycle motif. Our analysis sheds light on the regulatory strategies that safeguard inflammatory gene expression from overproduction and repositions the function of IκBα not only as a negative feedback regulator of NFκB but also as an enabler of NFκB-regulated stimulus-responsive inflammatory gene expression. This study indicates the complex involvement of IκBα in the inflammatory response to TNF that is induced by radiation therapy in breast cancer.

## Introduction

The phenotype of the cell is determined by the precise expression of its genes, which are regulated by transcription factors (TFs). Nuclear factor-κB (NFκB), a TF, is essential for regulating the transcription of immune response genes and cell death pathway-related genes^1–5^. The canonical NFκB signaling pathway is triggered by many inflammatory stimuli, including tumor necrosis factor (TNF), an important cytokine involved in chronic and acute inflammation^6^. Radiation therapy induces DNA damage^7^ and the release of TNF^8^, which induces apoptosis^9^ in cancer cells. Binding of TNF to its receptor activates NFκB and its target genes^2^.

NFκB consists of heterodimeric and homodimeric complexes of p65 (RelA), p50 (NFκB1), p52 (NFκB2), RelB, and c-Rel proteins^10–13^ and binds to the κB site, which is a 10 base pair consensus sequence^14^. There are three inhibitory κB (IκB) proteins (IκBα, IκBβ, and IκBε) that bind and retain NFκB in the cytoplasm. Upon TNF stimulation, they are phosphorylated and degraded, allowing NFκB to enter the nucleus, bind DNA, and activate transcription^15^. When all three IκB proteins are removed by mRNA knockdown or gene knockout, NFκB is unresponsive to TNF stimulation^16,17^. Thus IκB proteins are responsible for NFκB activation^18^. The three IκB proteins have distinct degradation and synthesis characteristics and therefore have distinct roles in shaping the dynamics of NFκB activity^2,19–21^. IκBα not only responds most rapidly to stimuli^15,22–24^ but also mediates a powerful negative feedback loop that may remove NFκB from DNA^25^ and result in oscillatory NFκB activity^15^. IκBβ and IκBε degrade more slowly upon TNF stimulation, and IκBε mediates a second negative feedback mechanism that functions in anti-phase to IκBα^24^. Thus, the precise balance of these IκB proteins determines the dynamics of nuclear NFκB activity in response to stimulation^2,19–21^.

Previous studies of IκBα’s effect on gene expression have focused on its role as a negative feedback regulator that limits the duration of NFκB activity when stimulated transiently, or that result in oscillations when stimulated with TNF for an extended period of time. In a transient stimulation protocol IκBα-deficient cells show enhanced expression of some genes^15^, as NFκB duration may be discriminated by a slow mRNA decay step or a slow chromatin step^26^. In the persistent stimulation protocol IκBα-deficient cells show NFκB-mediated eviction of many more nucleosomes and establishment of de novo enhancers^27^. However, whether the slowed and diminished NFκB activation observed in IκBα-deficient cells^15,24,28^ affects gene expression has not been examined.

Here we will address whether rapid NFκB activation by TNF, which is enabled by the degradation of IκBα within the IκBα-NFκB complex, is critical for activation of NFκB target genes in MCF7 breast cancer cells. Time-course NFκB activation and RNA-seq transcriptomic profiling data reveals a cohort of genes that are diminished in an siIκBα knockdown condition. By fitting mathematical models of alternative gene regulatory mechanisms (GRM) models to the data, we characterize the regulatory mechanism that render some genes sensitive to the presence of IκBα while others are not. Our study repositions the function of IκBα not only as a negative feedback regulator that may limit gene expression in some conditions but also as an enabler of NFκB-responsive inflammatory gene expression.

## Results

### NFκB target gene activation in the presence or absence of IκBα

**Fig. 1:**
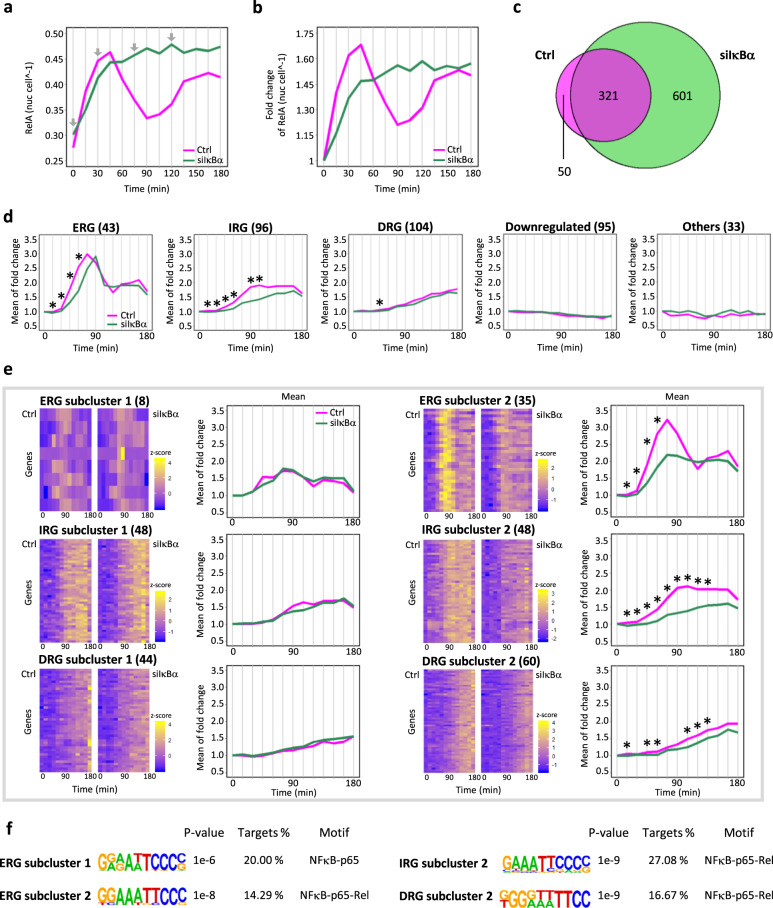
TNF (tumor necrosis factor)-induced RelA nuclear translocation and expression of NFκB target gene clusters. Time-course nuclear NFκB abundance (**a**) and fold change (**b**) from fixed-cell immunofluorescence of MCF-7 cells treated with 1 ng mL^−1^ TNF in Ctrl (Control) and siIκBα (IκBα knockdown). Line graphs represent the means of two biological replicates. Number of cells used to calculate the mean for each time point ranged from 1336 to 2374 cells (Supplementary Table 1). Gray lines indicate the time points where RNA-seq data was measured, and arrows indicate the time points where single-cell ATAC-seq data was measured. Statistical significance was observed for interpolated (same method used in mathematical modeling) time-course nuclear NFκB abundance (*p* value: 1.039e−4) and fold change (*p* value < 2.2e−16) between Ctrl and siIκBα from before stimulation and 75 min (the first peak time point of early response genes) after stimulation by one-tailed Wilcoxon rank-sum test. **c** Venn diagram of the TNF-induced DEGs in Ctrl and siIκBα. **d** Mean of fold change in the expression of five TNF-induced clusters (ERG, IRG, DRG, downregulated, and others) of DEGs in Ctrl. For these 5 clusters, statistical tests were performed for fold change in expression between Ctrl and siIκBα (*p value < 0.01 by one-tailed Wilcoxon rank sum test). **e** Two subclusters for each TNF-induced cluster (ERG, IRG, and DRG) in Ctrl (outlier *PCSK5* excluded in line graph). For these 6 subclusters, statistical tests were performed for fold change in expression between Ctrl and siIκBα (*p value < 0.01 by one-tailed Wilcoxon rank-sum test). **f** κB motifs that were enriched at promoter regions (±50 bps TSS) of ERG subclusters 1 and 2, IRG subcluster 2, and DRG subcluster 2.

To observe the difference in NFκB dynamics in the presence or absence of IκBα in MCF-7 cells, we conducted imaging experiments using fixed immunofluorescence to measure the time course of nuclear NFκB activity after stimulation with TNF (Supplementary Table 1). When IκBα was present, NFκB transiently translocated into the nucleus at earlier time points, followed by a dampened peak at later time points, whereas when IκBα was knocked down using small interfering RNA (siRNA), nuclear translocation of NFκB was prolonged (Fig. 1a). Furthermore, the basal level of nuclear NFκB abundance was 13% higher when IκBα was knocked down (Supplementary Fig. 1), and this difference in the basal level produced a significantly higher fold change in expression in the presence of IκBα (Fig. 1b).

To investigate how IκBα-regulated NFκB localization affects the expression of its target genes, we performed bulk RNA-sequencing at early time points (0–180 min) after stimulation with TNF in the presence (control) or absence of IκBα (siIκBα). We measured RNA levels every 15 min after stimulation to produce detailed expression patterns and identified 371 and 922 differentially expressed genes (DEGs) in control and siIκBα cells, respectively. We then compared the DEGs in each condition and identified 321 overlapping DEGs in both conditions (Fig. 1c). To observe the expression dynamics of DEGs in control cells, we performed Fuzzy *c*-means clustering and classified the 371 DEGs in control cells into five gene clusters: early response genes (ERGs), intermediate response genes (IRGs), delayed response genes (DRGs), downregulated, and others (Fig. 1d). To investigate genes that were differentially expressed in siIκBα but not in control, 601 DEGs that were unique to siIκBα were selected (Fig. 1c) and clustered into three groups: upregulated, downregulated, and others (Supplementary Fig. 2).

As the fold change in the expression level of a target gene is proportional to the fold change in the activity of the TF when they participate in an incoherent feedforward loop (IFFL)^29,30^, we statistically compared the fold change in expression between control and siIκBα for genes in each TNF-induced cluster. For the ERGs, IRGs, and DRGs, the fold change in gene expression at some time points were significantly higher in control cells (Fig. 1d). In contrast, the mean fold change in the expression of upregulated genes that were unique to siIκBα cells was consistently lower in control cells after stimulation (Supplementary Fig. 2). Furthermore, we investigated whether this gene cluster includes genes that are regulated by NFκB by performing motif analysis. This was performed at the promoter regions (±500 bps transcription start site (TSS)) of 195 protein-coding genes among 214 upregulated genes, but none of the NFκB subunit-binding motifs were significantly enriched (Supplementary Fig. 3). Results indicated that most TNF-induced DEGs that were upregulated only in siIκBα were unlikely to be controlled by NFκB.

To analyze the ERGs, IRGs, and DRGs in more detail, we further classified each cluster into two subclusters (Fig. 1e). For each gene cluster, genes in subcluster 1 showed a similar fold change in expression between control and siIκBα, and genes in subcluster 2 showed a statistically higher fold change in expression in the control than in siIκBα. We performed motif analysis at the promoter regions of each subcluster from the ERGs, IRGs, and DRGs and found that a subcluster 2 from all three showed a significantly enriched NFκB binding motif, while only subcluster 1 from the ERGs showed a significant enrichment (Fig. 1f). These data show that NFκB is involved in the induction of many TNF-responsive genes and indicate that IκBα functions not only as a negative feedback regulator^15,31^ of NFκB, but also as an enabler of NFκB-regulated stimulus-responsive gene expression.

### NFκB control of chromatin accessibility in the presence or absence of IκBα

To examine how NFκB-binding sites (κB sites) are affected by NFκB activation in the presence or absence of IκBα in individual cells, we performed a single-cell assay for transposase-accessible chromatin using sequencing (ATAC-seq) for early time points (0, 30, 75, and 120 min) after stimulation with TNF for control and siIκBα in MCF-7 cells. These time points were selected based on those that showed the maximum and minimum early time course (0–180 min) nuclear NFκB abundance (Fig. 1a). After sample curation, the remaining 989 and 953 single-cell samples in control and siIκBα, respectively, were aligned to the genome and their chromatin accessibility was quantified. Furthermore, we quantified the chromatin accessibility of the aggregated samples for each condition and identified the peak regions for each time point.

**Fig. 2:**
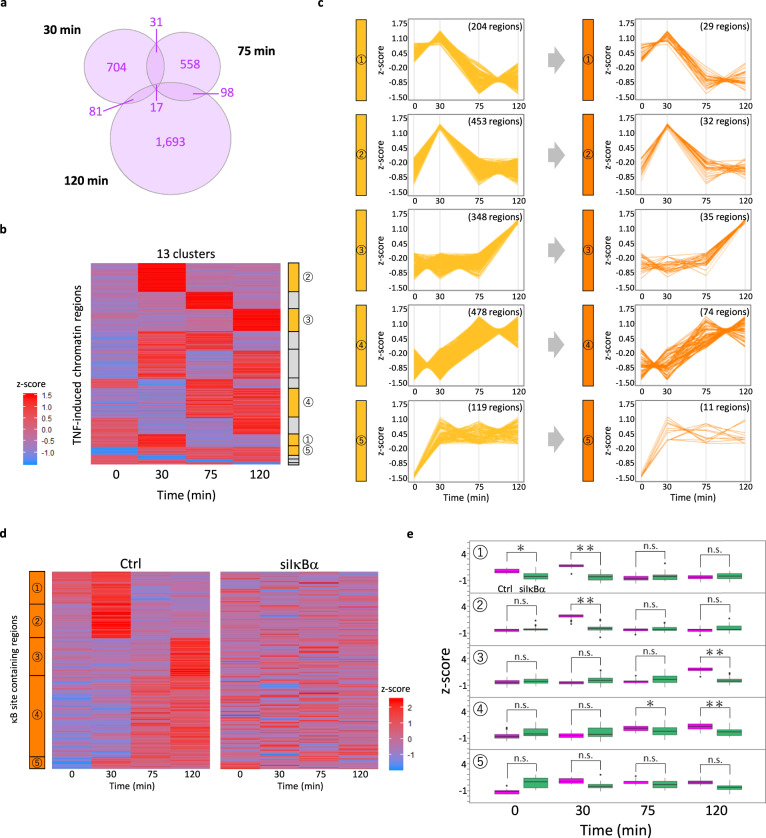
Dynamics of chromatin accessibility in κB sites after TNF (tumor necrosis factor) induction. **a** Venn diagram of the number of TNF-induced chromatin regions at each time point in Ctrl (Control). **b** Regions in **a** clustered into 13 time-course clusters. Chromatin accessibility was normalized to *z*-score, where red shows high chromatin accessibility and blue shows low chromatin accessibility in the heatmap. **c** Clusters enriched with κB sites and extracted κB site detected regions. Time-course chromatin accessibility was *z*-score normalized for each κB site-enriched region. Numbers in braces show the number of regions before and after κB site enriched regions were extracted for each cluster. **d** Time-course chromatin accessibility in Ctrl and siIκBα (IκBα knockdown) at κB site-detected regions in **c**. Chromatin accessibility was normalized to *z*-score, where red shows high chromatin accessibility and blue shows low chromatin accessibility in the heatmap. **e** Comparison of time-course chromatin accessibility between two conditions at κB site-enriched regions in **d**. Statistical tests were performed for chromatin accessibility between Ctrl and siIκBα at these regions in Ctrl and siIκBα (*p value < 0.01, **p value < 0.0001 and n.s.: *p* value ≥ 0.01 by one-tailed Wilcoxon rank-sum test). The center line indicates the median, the upper and lower hinges indicate the first and third quartiles, the upper whisker extends from the hinge to the largest value no further than 1.5× IQR (interquartile range) from the hinge, the lower whisker extends from the hinge to the smallest value at most 1.5× IQR of the hinge, and the points indicate the outliers.

Using these peak regions in the aggregated data, we identified peak regions of the ATAC-seq signal that were significantly induced at each time point after TNF stimulation. Within these 3643 regions in total, we filtered out regions that did not show higher chromatin accessibility after TNF stimulation than before. When the TMM normalization was applied to normalize the chromatin accessibility, we found that 833, 704, and 1889 regions were induced at 30, 75, and 120 min, respectively, in control cells (Fig. 2a). We then clustered all the 3182 regions based on their time-course chromatin accessibility. These regions were clustered into 13 groups based on their time-course patterns (Fig. 2b).

Next, we performed motif analysis at these TNF-induced ATAC peak regions in control cells to capture the trend of the time-course chromatin accessibility pattern of TNF-induced κB sites and to identify transcription regulators other than NFκB. There were five clusters that showed significant enrichment of κB sites (Fig. 2c), including binding sites for the interferon regulating IRF4^32^ and inflammatory cytokine regulating AP-1 subunit^33^ (Supplementary Fig. 4). We further extracted only the regions that were significantly enriched with κB sites from each of these clusters (Fig. 2c). The patterns of these extracted regions in each cluster reflected the nuclear NFκB abundance in control, showing a high chromatin accessibility at 30 and 120 min, which corresponds to the first and second peaks, respectively, of nuclear NFκB activity (Fig. 2d).

Similarly, we identified 3571 out of 4079 TNF-induced regions that were upregulated in siIκBα. These regions were clustered into 13 groups (Supplementary Fig. 5), and motif analysis was performed to capture the trend of time-course chromatin accessibility. We obtained five clusters that showed significant enrichment of κB sites, including IRF4- and AP-1-binding sites (Supplementary Fig. 5). Then we extracted only the regions that were significantly enriched with κB sites from each of these clusters (Supplementary Fig. 6). The time-course patterns of these extracted regions in each cluster reflected the nuclear NFκB abundance in siIκBα, showing high and prolonged chromatin accessibility at late time points (Supplementary Fig. 7).

To confirm the statistical difference between chromatin accessibility at the TNF-induced clusters in the presence and absence of IκBα, we calculated the aggregated time-course chromatin accessibility for each cluster in control and siIκBα cells and performed a one-tailed Wilcoxon rank sum test between the two conditions for each cluster at each time point (Fig. 2d, e and Supplementary Fig. 7). Furthermore, to investigate whether the individual cells in control and siIκBα can be distinguished by chromatin accessibility at these TNF-induced regions between the presence and absence of IκBα, we performed 10-fold cross-validation using Bayesian generalized linear model.

As a result, substantial statistical significance (*p* value <0.0001) from aggregated cells and classification accuracy of >60% from single cells were observed at 30 and 120 min of TNF-induced regions in the presence of IκBα, which corresponded to the peaks of nuclear NFκB abundance in control (Fig. 2d, e, cluster 2 at 30 min and cluster 3 at 120 min). However, TNF-induced regions in the absence of IκBα showed these statistical significance and classification accuracy at only late time points (Supplementary Fig. 7, cluster 1 and 5 at 75 min and cluster 4 at 120 min). These results suggest that IκBα is responsible for the rapid NFκB activation dynamics that allows NFκB to mediate early chromatin activation.

### A simple mathematical model accounts for many DRGs in subcluster 2

The expression patterns of ERGs, IRGs, and DRGs in subcluster 2 in control cells followed a similar pattern with the time-course nuclear NFκB dynamics. However, whereas nuclear NFκB activity time course showed a prolonged pattern in siIκBα, many ERGs showed post-induction repression. This implied that there is a post-induction repression mechanism of transcriptional control that is independent of IκBα.

To unravel this mechanism, we first applied a simple mathematical model to the data on the nuclear NFκB activity and its target genes to identify the regulatory mechanisms (detailed description in “Methods”). To recapitulate the time-course fold change in expression observed in the RNA-seq data, we calculated the fold change of input nuclear NFκB activity and scaled it to avoid assay-specific reductions in the dynamic range for two biological replicates of NFκB translocation data (Supplementary Fig. 8). We optimized the parameters of the model for each biological replicate and identified parameter sets that were the most concordant among the 10,000 pairs of two biological replicates according to the Pearson correlation coefficients (Supplementary Fig. 9) to justify each parameter value. We simulated fold change in expression using these concordant parameter sets and examined whether the model was acceptable for each gene. To examine this, we set a definition referring to the results of the RNA-seq data analysis (Fig. 1e). First, to be a good fit, the nRMSD (normalized root mean square deviation) values in replicates 1 and 2 of the control set should be <0.5, and the nRMSD values in replicates 1 and 2 of the siIκBα should be <0.39. In addition, since all ERGs, IRGs, and DRGs in subcluster 2 showed a larger value in the control than in siIκBα for at least either their max-fold induction (MFI) or the area under the curve (AUC), we defined that at least either the simulated MFI or AUC should also show the same relationship (control > siIκBα) to be a good fit (Supplementary Fig. 10).

In subcluster 2, there were 5 out of 34 ERGs, 19 out of 48 IRGs, and 50 out of 60 DRGs that showed a good fit (Supplementary Fig. 10). Many DRGs showed a good fit with this model, and the fold change in expression was similar between control and siIκBα, showing a monotonically increasing expression pattern. Representative good-fit DRGs included *LTB*, which encodes a membrane protein that promotes inflammation through the activation of the NFκB signaling pathway^34^, and *RELB*, which encodes a protein that is a subunit of NFκB complex that is involved in immune tolerance to inflammation^35^. However, this model failed to recapitulate the patterns of many other in subcluster 2, including ERGs in subcluster 2 which showed post-induction repression. These results implied additional GRMs.

### The IFFL model accounts for reduced expression in siIκBα

Since the simple model was insufficient to describe the post-induction repression mechanism, we turned to a previously introduced IFFL model^29^. The IFFL model detects the fold change in the nuclear NFκB abundance and reflects this change on gene expression. This model demonstrates a NFκB-regulated competitor TF that competitively binds to the NFκB target gene promoter region. From among the ERGs, IRGs, and DRGs, we searched for TNF-induced DEGs, which are also NFκB target genes, for possible NFκB competitors^29^. We identified the *NFKB1* encoded protein, which is processed into p50 as the only competitor. We defined the processing time of p50 from information gathered from previous studies and fixed the time between nuclear p50 translocation and mature poly A^+^ mRNA production as 2 h^36,37^ (detailed description in “Methods”). After fixing the processing time, we applied this model to the ERGs, IRGs, and DRGs in subcluster 2 by using the same parameter optimization flow, identification process of concordant parameter sets (Supplementary Fig. 11), and the definition of the good fit as those used for the simple model analysis.

In subcluster 2, there were 5 out of 34 ERGs, 5 out of 48 IRGs, and 23 out of 60 DRGs that showed a good fit (Supplementary Fig. 12). The good fit genes in each cluster showed a reduced fold change in expression in siIκBα, similar to the nuclear NFκB activity fold change in siIκBα, which showed a lower fold change level than in control. The IFFL model detects the nuclear NFκB activity fold change, and thus the fold change in gene expression closely follows this reduction in siIκBα. However, the transcription regulatory mechanisms for most ERGs and IRGs in subcluster 2 were not described by this model (Supplementary Table 2).

### A 3-state cycle model accounts for post-induction repression

The IFFL model was able to describe the reduced fold change in expression in siIκBα, but since many ERGs showed post-induction repression, we hypothesized a mechanism that physically inhibits the binding of NFκB after transcription inhibition. This encouraged us to construct a model that describes the transition of the promoter state. This model consists of nuclear NFκB, closed promoter state that is driven to an open promoter state, and the active promoter state that is driven by the open promoter state. The active promoter state is refractory and is driven to a closed promoter state (note that the rate constant of the regulation from the active state to the closed state may result from a combination of several transcriptional mechanisms, including the dissociation of NFκB). The active state also drives transcription, which includes the synthesis and degradation of mRNA. There are backward reactions between this closed chromatin state and open chromatin state, as well as between the open chromatin state and active chromatin state (detailed description in “Methods”). We hypothesized that post-induction repression occurs when the promoter state of a target gene changes from active to closed after nuclear NFκB translocation.

We applied this model to the ERGs, IRGs, and DRGs in subcluster 2 by using the same parameter optimization flow and the definition of a good fit as the simple model analysis. When fold change in expression was simulated with the concordant parameter set (Supplementary Fig. 13) for each gene in subcluster 2, there were 7 out of 34 ERGs, 24 out of 48 IRGs, and 55 out of 60 DRGs that showed a good fit (Supplementary Fig. 14). A representative IRG, *BCL2L11*, which encodes a protein that functions as a tumor suppressor by inducing apoptosis^38^, showed a good fit with this model. However, despite the presence of many ERGs in subcluster 2 that showed post-induction repression similar to the good fit genes in other clusters, there were only a few ERGs that showed a good fit with this model (Supplementary Fig. 14). This was because many of the ERGs in subcluster 2 that showed post-induction repression also showed reduced expression in siIκBα.

### A combined model v4 accounts for both post-induction repression and reduced expression

Finally, since many ERGs showed both post-induction repression and reduced expression in siIκBα, we constructed a model that combines the 3-state cycle model and the IFFL model by applying the previous three models. In this model, the NFκB competitor suppresses the NFκB target gene promoter in the active state, which suppresses transcription of the target gene (detailed description in “Methods”). We hypothesized that by adding the IFFL model to the 3-state cycle model, both post-induction repression and reduced expression can be observed at the same time.

We applied this model to the ERGs, IRGs, and DRGs in subcluster 2 by using the same parameter optimization flow and the definition of the good fit as the simple model analysis. When fold change in expression was simulated with the concordant parameter set (Supplementary Fig. 15) for each gene in subcluster 2, there were 13 out of 34 ERGs, 12 out of 48 IRGs, and 38 out of 60 DRGs that showed a good fit (Supplementary Fig. 16). This model was able to recapitulate many ERGs in subcluster 2, which showed both post-induction repression and reduced expression, indicating that the transition of promoter states and the suppression of transcription at the NFκB target gene promoter by the competitor protein are both required. In particular, inflammation and cancer-related ERGs (e.g., *A20* and *JUNB*) showed a transient fold change in expression. Overproduction of these genes results in inflammation, metastasis, invasion, and hormone-resistant phenotypes^33,39–41^. Among these genes, JUNB is a TF that induces pro-inflammatory cytokines (e.g., TNF, interleukin (IL)-6, and IL-12)^33^. TNF and IL-6 possess both antitumor and protumor properties^42–45^, whereas IL-12 promotes inflammation during the antitumor immune response^46,47^. The post-induction repression of these mechanisms functions as a brake for possible aberrant transcription induction of these cytokine-regulating TFs, which in turn safeguards against the overproduction of cytokines with protumor (e.g., TNF and IL-6) and excessive inflammation (e.g., IL-12) activities. While this repression acts as a brake for tumor progression and aberrant inflammation for some genes, it also acts as a suppressor of apoptosis, as observed for *KLF10*^48^. Given that apoptosis suppresses tumor progression, these contradictory effects are controlled by a post-induction repression mechanism that safeguards gene expression from overproduction.

### Identification of the best-fit model for each ERG, IRG, and DRG in subcluster 2

We applied four mathematical models to each ERG, IRG, and DRG in subcluster 2 to identify the transcription mechanism of TNF-induced gene expression. We first applied a simple model that considers only the relationship between the fold change in nuclear NFκB activity and the transcription of its target gene. This model recapitulated many DRGs in subcluster 2, which showed a similar level of fold change in expression between control and siIκBα, and a monotonically increasing expression pattern. However, most of the ERGs in subcluster 2 did not fit well with this model. Next, we applied the IFFL model, which is a detectable fold-change model. Since the fold change in nuclear NFκB is higher than that in siIκBα, reduced expression in siIκBα was recapitulated by this model. A few ERGs in subcluster 2 showed a good fit with this model, but many genes did not. To recapitulate post-induction repression, we constructed a 3-state cycle model and identified good fit genes. This model was able to recapitulate post-induction repression but not reduced expression in siIκBα. Therefore, we constructed model v4, which combines the 3-state cycle and IFFL model. This model was able to recapitulate both post-induction repression and reduced expression in siIκBα for many ERGs in subcluster 2.

**Fig. 3:**
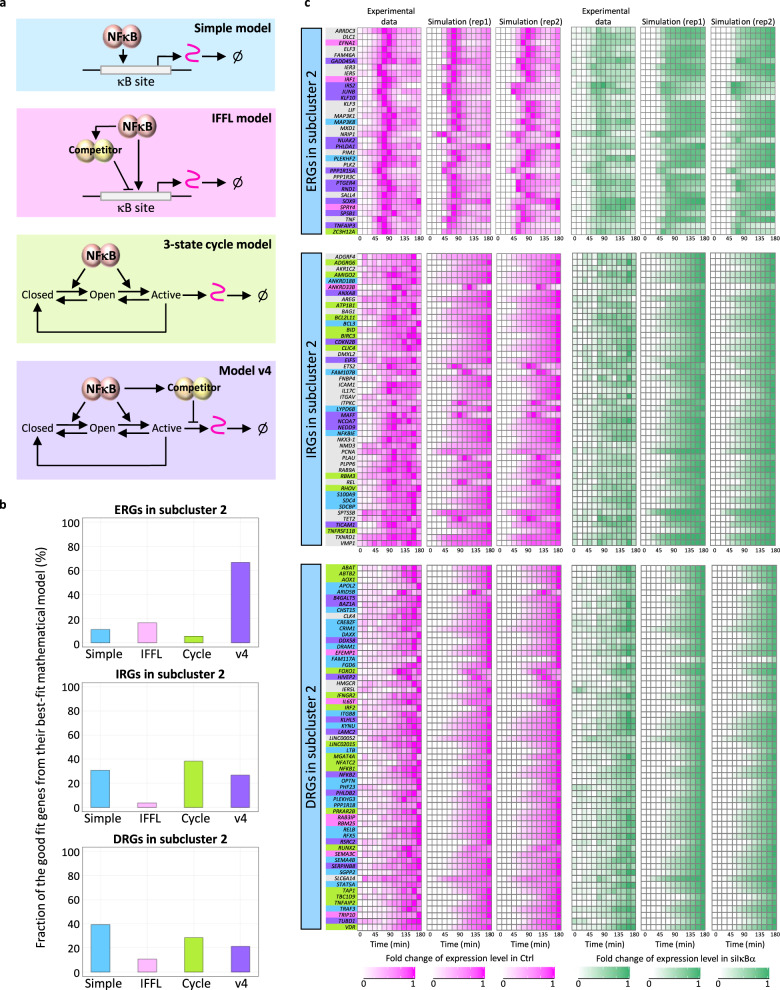
Model v4 recapitulated fold change in expression of many early response genes in subcluster 2. **a** Schematic of the four mathematical models. The color code corresponds with the background color of each model. **b** Fraction of the good-fit genes from their best-fit mathematical models. **c** Heatmaps of the time-course gene expression from experimental results and data fit from the best-fit model. Each color bar indicates the best-fit model (blue: simple model, magenta: IFFL model, green: 3-state cycle model, and purple: model v4), which shows the smallest total nRMSD (nRMSD in Ctrl from rep1 + nRMSD in siIκBα from rep1 + nRMSD in Ctrl from rep2 + nRMSD in siIκBα from rep2). Gray colored bars indicate genes that did not show a good fit with any of the four mathematical models, and the results from the simple model are shown.

Since there were genes that showed a good fit with multiple mathematical models, we identified the best-fit model for each gene (Fig. 3a). For each, we calculated the total nRMSD in replicate 1, nRMSD of siIκBα in replicate 1, nRMSD in replicate 2, and nRMSD of siIκBα in replicate 2 for each model, which showed a good fit. The total nRMSDs were then compared between the good-fit models, and the model with the smallest total nRMSD was identified. This model was defined as the “best-fit” model for each good-fit gene. We found that the best-fit model of many ERGs was model v4 (Fig. 3b, c), showing a transient expression pattern (Supplementary Table 3). Interestingly, among the ERGs recapitulated with model v4, there were well-known NFκB pathway regulators^40,41^ (e.g., A20) and TFs (e.g., JUNB and KLF10), which are key inflammation and breast cancer regulators^33,39,48^. For the IRGs, *BCL2L11* encoded protein, known as a tumor suppressor^38^, was recapitulated by the 3-state cycle model, showing a monotonically increasing expression pattern (Supplementary Table 4). Among the DRGs, the *LTB* encoded membrane protein, known as TNF C, which promotes inflammation through the activation of the NFκB signaling pathway^34^, and the *RELB*, known to be involved in immune tolerance to inflammation and to repress proinflammatory genes^35^ were recapitulated by the simple model (Supplementary Table 5), showing a monotonically increasing expression pattern.

### Chromatin remodeling at κB sites in promoter regions of post-induction repressed genes

To confirm that transcription of post-induction repressed ERGs in subcluster 2 that were best demonstrated by the 3-state cycle model or model v4 are regulated by chromatin remodeling, we investigated the time-course chromatin accessibility at the κB sites in the promoter regions of these genes using aggregated single-cell ATAC-seq data. First, we identified κB site-enriched ATAC-seq peak regions at each time point in control and siIκBα for each subcluster 2. All 3 subclusters showed more κB site-enriched peak regions in siIκBα than in control (Supplementary Fig. 17).

However, none of these κB site-enriched peak regions were included in the κB site-enriched peak regions identified in Fig. 2. This was because we focused on only significantly induced peak regions after stimulation in Fig. 2, whereas most of the peak regions were not significantly induced but were induced to some extent during the time course. Thus, from these κB site-enriched peak regions in promoter regions of genes in subcluster 2, we further extracted only the κB site-containing regions at each time point and merged these regions for multiple time points. The time-course expression of genes in subcluster 2 showed their peak at 75 min for ERGs, 75 min and 120 min for IRGs and DRGs (Supplementary Fig. 18). Therefore, we expected that κB sites in promoter regions of these genes will show a start of decrease in chromatin accessibility at least at 30 or 75 min in both control and siIκBα.

**Fig. 4:**
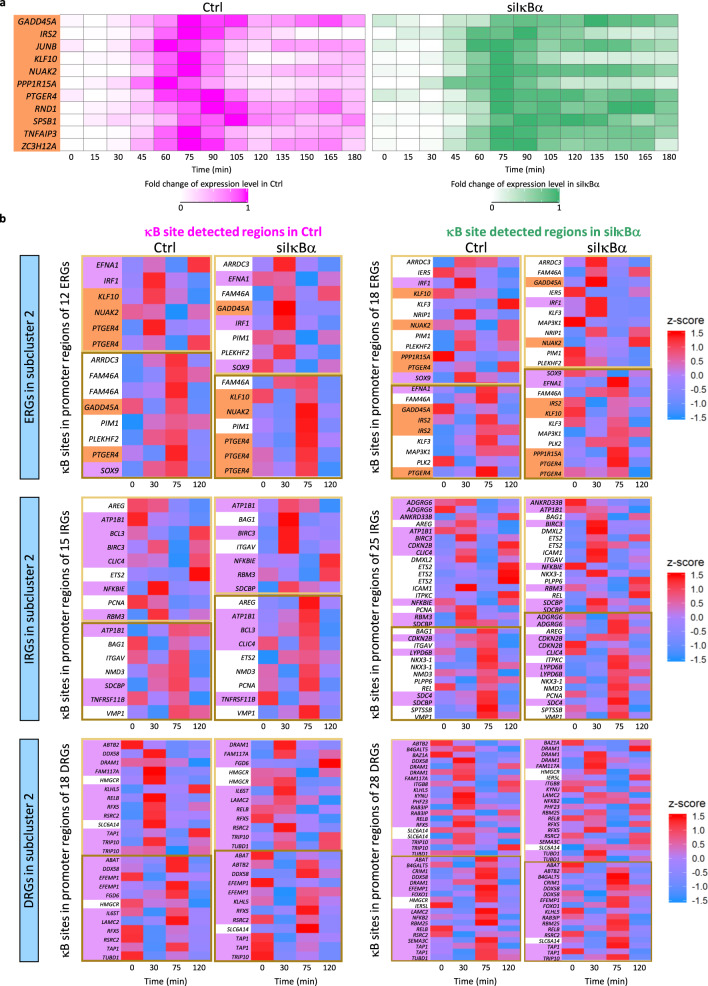
Chromatin remodeling observed for many post-induction repressed ERGs in subcluster 2. **a** Heatmaps of time-course fold change in expression from data of all post-induction repressed genes that were demonstrated by the 3-state cycle model or model v4 (highlighted in orange). Genes shown in the heatmaps all belong to the ERGs in subcluster 2. **b** Heatmaps of time-course chromatin accessibility at κB sites in promoter regions of ERGs, IRGs, and DRGs in subcluster 2, which showed the start of decrease in chromatin accessibility at least at 30 (light brown box) or 75 (brown box) min in both Ctrl and siIκBα. Genes highlighted with orange are post-induction repressed ERGs in subcluster 2 that were best demonstrated by 3-state cycle model or model v4. Genes highlighted with purple are genes that were demonstrated by any of the 4 models but did not show post-induction repression in data.

We identified κB site-detected regions that showed these patterns and found that more than 50% of the post-induction repressed ERGs in subcluster 2 that were demonstrated by 3-state cycle model or model v4 (6 out of 11 genes) showed a decrease in chromatin accessibility before or at the same time (75 min) when post-induction repression was observed (Fig. 4a, b). These included the *KLF10*, which is a key inflammation-related TF that is an apoptosis-inducing tumor suppressor^48^. In contrast, less than 50% of the ERGs, IRGs, and DRGs in subcluster 2 demonstrated by any of the 4 models (41 out of 89 genes) that did not show post-induction repression showed these patterns (Fig. 4b). These results suggest that many post-induction repressed ERGs in subcluster 2 showed chromatin remodeling at their promoter regions while many others did not, and these findings are consistent with the results obtained from mathematical modeling, showing that the transcriptional regulation of these post-induction repressed genes were best demonstrated by the GRMs, which include chromatin remodeling modules.

## Discussion

Previous studies have focused on IκBα’s role as a negative feedback regulator of NFκB activity, which limits the duration when stimulated transiently^15,28^. Thus, in IκBα-deficient cells, transient stimulation results in prolonged NFκB activation^15,28^, which in turn induces enhanced expression of some genes^15^. It was shown that prolonged NFκB activity allows genes to be regulated by a slow chromatin step or by slow mRNA decay to be activated more fully^26^. Persistent stimulation may result in NFκB oscillations mediated by the IκBα feedback loop; in their absence NFκB was shown in macrophages to generate hundreds of de novo enhancers by triggering nucleosome eviction^27^, suggesting that IκBα’s role is to preserve the enhancer landscape. However, the absence of IκBα also results in slowed and diminished activation of NFκB, as other IκB family members have slower IKK-responsive degradation kinetics^24^. Whether IκBα’s role to provide rapid NFκB activation is important for gene activation have not been examined.

Physiologically, radiation is known to induce apoptosis^49^ through the expression of TNF^8^, which is also known to inhibit cell proliferation^50^. TNF activates the canonical NFκB signaling pathway, which induces IκBα degradation and releases NFκB to the nucleus for transcriptional regulation^2^. We confirmed the expression of a gene that is involved in immune tolerance to inflammation^35^ (e.g., *RELB*) and apoptosis-inducing tumor-suppressor genes^38,48^ (e.g., *BCL2L11* and *KLF10*). However, the expression of inflammation and cancer-progressing genes^33,34,39–41^ (e.g., *A20*, *JUNB*, and *LTB*) were also observed when IκBα was present, indicating that IκBα enables the full induction of not only NFκB-regulated genes that promote apoptosis, but also NFκB-regulated genes that promote inflammation and cancer progression. Here, our findings provided by studying gene expression in cells that contain IκBα and those that do not suggest that IκBα not only functions as a negative feedback regulator, but also as an enabler of some NFκB-regulated stimulus-responsive inflammatory gene expression and NFκB-regulated early chromatin activation. This indicates the complex involvement of IκBα in NFκB transcription regulation, activated by TNF.

Secondly, we explored how the altered dynamics in IκBα deficient cells are interpreted by transcription regulatory mechanisms of TNF-induced NFκB target genes. In particular, expression of some ERGs was repressed in the absence of IκBα after induction. Given that the time course of nuclear NFκB activity in the absence of IκBα was prolonged, these results indicated the existence of GRMs. We investigated these mechanisms by fitting GRM models to the RNA-seq transcriptomic profiling data.

**Fig. 5:**
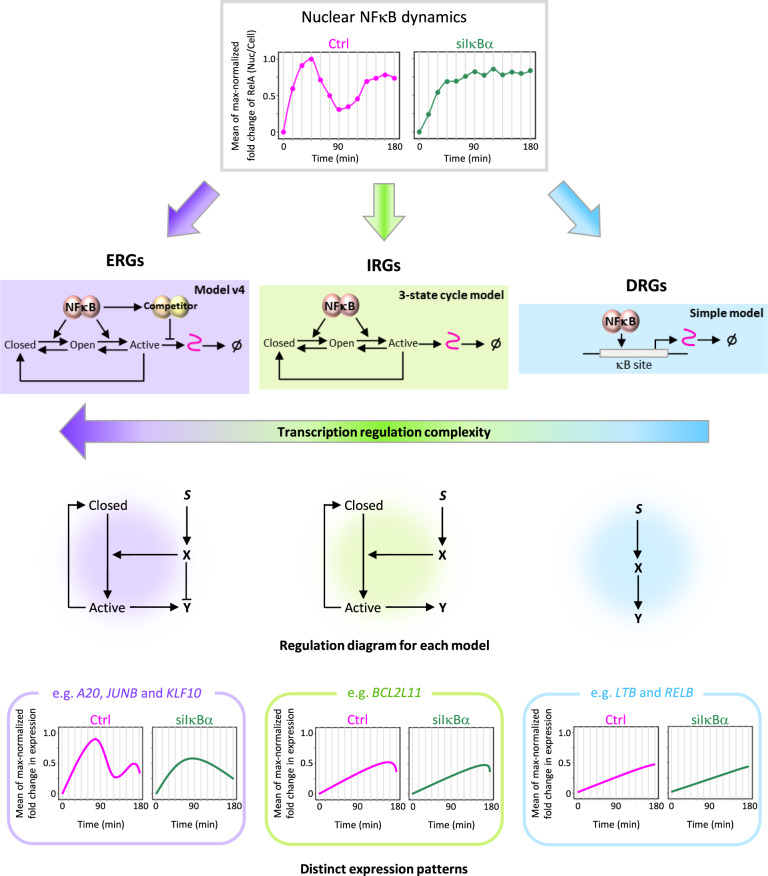
Distinct regulatory mechanisms of NFκB target genes depending on their response time. Diagram of the distinct transcription regulatory mechanisms that characterizes each TNF-induced NFκB target gene cluster. Mean fold change in nuclear NFκB abundance of two biological replicates in Ctrl and siIκBα were interpolated to every second (same method used in mathematical modeling) and were max-normalized from 0 to 1 together, shown as line graphs in the top panel. The dots indicate the data from fixed-cell immunofluorescence experiments. The bottom panel shows the schematic curves of the expression patterns of ERGs, IRGs, and DRGs in subcluster 2. Despite the persistent pattern of nuclear NFκB abundance in siIκBα, post-induction repression was observed for the ERGs, and model v4 was the most effective model to recapitulate post-induction repression and reduced expression. For the IRGs, the 3-state cycle model was the most effective model, and for the DRGs, the simple model was the most effective model. The complexity of the transcription regulatory mechanisms increases as the response time of transcription decreases. Inflammation and cancer-related *JUNB* and *KLF10*, respectively, showed a transient fold change in expression, indicating that post-induction repression mechanism safeguards gene expression from overproduction, protecting from inflammatory diseases and tumor progression.

While the NFκB activation mechanism is common between cells, basal nuclear NFκB activity varies from cell to cell because of heterogeneity in protein expression^51,52^ and kinase activity. A previous study revealed that fold change of NFκB activity rather than absolute NFκB abundance in HeLa cells provided a more statistically robust explanation for the observed variability in expression between cells^29^. The fold-change detection mechanism provides an analog of Weber’s law, which discriminates the signal relative to the background signal^53^. In signaling systems, it may be mediated by an IFFL, in which a TF regulates its target gene and a repressor of the target gene. Since the fold change detection mechanism in the transcription of target genes is introduced by an IFFL of human cells^54–56^ and is also found in the NFκB signaling pathway, we applied the IFFL model. Consequently, we found that a combined model that inserts the IFFL model into the 3-state cycle model recapitulated many ERGs, indicating that transcription is regulated by a post-induction repression mechanism that drives the promoter state from active to closed and a detectable fold change mechanism that renders these genes sensitive to the presence of IκBα. As a whole, the complexity of the transcription regulatory mechanism increased as the response time to TNF stimulation decreased, indicating that inflammation and cancer-related ERGs require a strict and precise transcriptional regulation to avoid overproduction (Fig. 5).

The post-induction repression can be explained by molecular mechanisms that involve chromatin remodeling complexes that have been revealed in previous studies^57,58^. For example, the nucleosome remodeling and deacetylation (NuRD) complex is recruited to its target sites by transcriptional repressors and/or methylated DNA, and nucleosome remodeling facilitates histone deacetylation by HDAC1/2 subunits of the NuRD complex^57^. This enzymatic reaction promotes the folding of chromatin into a repressed, higher-order structure, which in turn leads to the loss of RNA polymerase II and represses transcription of some genes^57^. At the same time, resetting the local nucleosome landscape and initializing the TSS of RNA polymerase II establishes a new transcriptional state, where some genes show an overall increase in expression levels^59^. In addition, NuRD complex is known to promote the activity of an another chromatin remodeling complex called the polycomb repressive complex 2 (PRC2)^60,61^, which initially targets the genomic region for chromatin remodeling by methylating H3K27. H3K27me3 produced by PRC2 acts as a docking site for the PRC1 to induce chromatin compaction, which reduces not only the accessibility of TFs^58^ but also the ATP-dependent chromatin-remodeling machineries, such as the SWI/SNF complex^62^. The SWI/SNF complex also catalyzes ATP-dependent chromatin remodeling by coupling ATP hydrolysis with directional movement over DNA, which represses transcription of some genes^63^. Chromatin remodeling mechanisms including these mechanisms may cooperatively or independently induce the post-induction repression observed for many ERGs found in this study.

This study provides insights that may be used to reveal the transcription regulatory mechanisms in many biological systems, such as systems that require both post-induction repression and fold change detection. TNF-induced NFκB target genes that were recapitulated by either of the four models are likely robust to noise, because the models applied here were all deterministic. In addition, genes that did not show a good fit when either of the four models were applied may be regulated by an additional combination of a transcription regulatory model, such as the existence of multiple repressors and activators^64–67^. Other factors may also affect the transcription of target genes. For example, the dose of the TNF stimulus changes the NFκB dynamics, which in turn alters the transcription pattern of the target gene^68^, or the abundance of the different NFκB dimers affects the transcription state of the target gene^69^. Transcription regulation is affected by various factors and is controlled by a sensitive balance in each cell.

In summary, we found that rapid IκBα-mediated NFκB activation is required for full induction of some NFκB-regulated target genes and for NFκB-regulated early chromatin activation. Among the TNF-induced target genes that showed reduced fold change in expression in the absence of IκBα, we characterized GRMs that render some genes sensitive to the presence of IκBα by fitting GRM models to data. Our study repositions the function of IκBα not only as a negative feedback regulator but also as an enabler of NFκB-regulated stimulus-responsive inflammatory gene expression in the signaling pathway and proposes GRMs that safeguard inflammatory gene expression from overproduction.

## Methods

### MCF-7 cell culture and TNF treatment

The human breast adenocarcinoma MCF-7 cell line was purchased (American Type Culture Collection, Manassas, VA, USA) and propagated in Dulbecco’s modified Eagle’s medium (Thermo Fisher Scientific, Waltham, MA, USA), supplemented with 10% fetal bovine serum (FBS) and antibiotics (100 units mL^−1^ penicillin and 100 μg mL^−1^ streptomycin, Nacalai Tesque, Kyoto, Japan). TNFα (Thermo Fisher Scientific) was dissolved in a 0.1% bovine serum albumin/phosphate-buffered saline (PBS) solution at a concentration of 100 ng mL^−1^ and added to the cells at a final concentration of 1 ng mL^−1^.

### siRNA transfection

Reverse transfection was performed using Hiperfect reagent (Qiagen, Hilden, Germany) according to the manufacturer’s instructions. Trypsinized MCF-7 cells were resuspended in an antibiotic-free medium and then mixed with a suspension of Opti-MEM (Thermo Fisher Scientific) containing 50 nM siRNA and Hiperfect reagent in 60-mm dishes (for ATAC-seq), 6-well plates (for RNA-seq), or 96-well plates (for immunostaining). SMARTpool ON-TARGETplus siRNA targeting IκBα (L-004765-00, a mixture of four sequences: AGUCAGAGUUCACGGAGUU, GCUGAUGUCAACAGAGUUA, AGGACGAGCUGCCCUAUGA, GUGCUGAUGUCAAUGCUCA) and ON-TARGETplus Non-targeting siRNA (D-001810-02, UGGUUUACAUGUUGUGUGA) were purchased from Dharmacon (GE Healthcare, now Horizon Discovery, UK). siRNA transfections were carried out 3 days before TNFα stimulation.

### Immunostaining

The method of immunostaining and quantification of signal intensity at every single cell was slightly modified from the previously reported method^70^. The control and IκBα-knockdown MCF-7 cells were seeded at a density of 1 × 10^4^ cells well^−1^ in 96-well plates. Cells were exposed to 1 ng mL^−1^ of TNFα for 0–3 h at 15 min intervals, fixed with 4% paraformaldehyde (Electron Microscopy Science, Hatfield, PA, USA) in PBS for 15 min, permeabilized with 0.1% Triton X-100 in PBS for 5 min, and washed with PBS. After incubating with blocking solution (10% FBS in Blocking One, Nacalai Tesque) for 1 h at room temperature, the cells were exposed to anti-RelA antibody (#8242, Cell Signaling Technology, Danvers, MA, USA) diluted 1:200 with by blocking solution at 4 °C. The next day, the cells were stained with Dylight550 anti-rabbit-IgG antibody (#84541, Thermo Fisher Scientific) diluted 1:500 with blocking solution for 1 h at room temperature, and thereafter stained with 0.2 mg mL^−1^ 4,6-diamidino-2-phenylindole (DAPI) in PBS for nuclei detection. Fluorescence images were obtained by using InCell Analyzer 2000 (GE Healthcare, now Cytiva, Marlborough, MA, USA). The Developer Toolbox software (Cytiva) was used to segment area of cells from bright-field images, segment area of nuclei from DAPI images, and quantify signal intensities for each cell. The nuclear-to-cell signal ratios were calculated based on the integrated signal density (i.e., mean signal intensity × area) for two biological replicates.

### RNA extraction and mRNA sequencing

The control and IκBα-knockdown MCF-7 cells were exposed to 1 ng mL^−1^ of TNFα for 0–3 h at 15 min intervals. Duplicate samples were used for total RNA extraction with NucleoSpin RNA Plus (Takara Bio, Shiga, Japan) according to the manufacturer’s instructions. The concentration and integrity of RNA were evaluated using the Bioanalyzer 2100 (Agilent Technologies, Santa Clara, CA, USA). Thereafter, library preparation was performed using 50 ng of mRNA with the Sureselect Strand RNA Kit (Agilent Technologies) according to the manufacturer’s protocol. A 36 bp single-read was performed using the HiSeq2500 System (Illumina, San Diego, CA, USA) with distinct samples from the immunostaining experiments.

### Single-cell ATAC-seq

To investigate whether the expression of NF-κB target genes was affected by chromatin accessibility, single-cell ATAC-seq was performed using the control and IκBα-knockdown MCF-7 cells for three biological replicates (distinct samples from immunostaining experiments and bulk RNA-sequencing experiments were used). The cells were stimulated with 1 ng mL^−1^ TNFα for 0, 30, 75, and 120 min and then collected using TrypLE™ Select (Thermo Fisher Scientific). The cells were washed twice with ice-cold PBS. The cell aggregates were removed by the pluriStrainer 20 μm (pluriSelect Life Science, Leipzig, Germany) and resuspended in PBS (250 cells μL^−1^). The preparation of single-cell ATAC-seq libraries using the C1 system (Fluidigm, South San Francisco, CA, USA) and Nextera DNA Library Preparation Kit (Illumina) was performed with reference to the previously reported method in the paper^71^ and the deposited protocol in the manufacturer’s platform (Fluidigm script Hub https://www.fluidigm.com/c1openapp/scripthub/script/2015-06/single-cell-chromatin-accessib-1433443631246-1, Revision C), with some modifications: The cell suspension and suspension regents (Fluidigm) were mixed in a 7:3 ratio and loaded into a C1 Single-Cell Open App IFC 17–25 μm (Fluidigm). Thereafter, a phase-contrast microscope was used to check whether any single cell without cell debris was captured. The cells were lysed and exposed to ATAC reaction by Tn5 transposition mix [1.5× TD buffer, 1.5× Tn5 transposase (Nextera DNA Sample Prep Kit, Illumina), 1.5× C1 Loading Regent with no salt (Fluidigm), 0.15% NP-40] at 37 °C for 30 min. Tn5-DNA complexes were dissociated from chromatin via the addition of the EDTA buffer (50 mM EDTA, 8.5 mM Tris-HCl pH 8, 1× C1 Loading Regent with no salt) for 30 min at 50 °C, and thereafter free EDTA was quenched by the MgCl_2_ buffer (45 mM MgCl_2_, 0.5 mM Tris-HCl pH 8, 1× C1 Loading Regent with no salt). Then polymerase chain reaction (PCR) was performed with ATAC Seq PCR Mix [1.4 μM non-indexed custom Nextera PCR primers 1 and 2 (Illumina), 1× C1 Loading Reagent with low salt, and 1.1× NEBnext High-Fidelity PCR Master Mix (New England Biolabs, Ipswich, MA, USA)] using the following conditions: 72 °C for 5 min; 98 °C for 30 s; and thermocycling at 98 °C for 10 s, 72 °C for 30 s, and 72 °C for 1 min. The amplified transposed DNA in every single cell was collected in approximately 3.5 μL each of C1 Harvest Reagent each (Fluidigm). The single-cell DNA library was collected from C1 Single-Cell Open App IFC (Fluidigm) and mixed with 10 μL of C1 DNA Dilution Reagent (Fluidigm). To dual-index the harvested libraries, 10 μL of harvested libraries were amplified for an additional 14 cycles in 50 μL of PCR reagent [1.25 μM custom Nextera dual-index PCR primers (Illumina) in 1× NEBnext High-Fidelity PCR Master Mix (New England Biolabs)] with the following PCR conditions: 72 °C for 5 min; 98 °C for 30 s; and thermocycling at 98 °C for 10 s, 72 °C for 30 s, and 72 °C for 1 min. The PCR products from 96 single cells were collected in a single tube and purified using a single MinElute PCR Purification Kit column (Qiagen). The purified library was then eluted with 20 μL of pure H_2_O. To remove primer dimers, the pooled libraries were purified twice or thrice using the same volume of AMPure XP beads (Beckman Coulter, Brea, CA, USA). After quantifying the library using the Bioanalyzer 2100, multiplex sequencing (36 bp single-read) was performed using HiSeq2500 (Illumina).

### Sequencing mapping of bulk RNA-seq data

Bulk RNA-seq datasets were aligned using HISAT2^72^ version 2.0.5 to build version “GRCh38/hg38” of the human genome after adapter trimming by TrimGalore^73^ version 0.6.0. Alignments were performed using option “–p 12 –q” where –p 12 indicates that 12 threads were ran on parallel processors and synchronized when parsing reads and outputting alignments; –q indicates that the reads are in FASTQ file format. Mapped reads assigned to genomic features were counted using featureCounts^74^ version 1.5.2. Assignments were performed using the option “-t exon –g gene_name” where -t exon indicates that featureCounts^74^ specifies the feature type in “exon”; and –g gene_name indicates that gene_name is the attribute type used to group features when GTF annotation is provided.

### Quantification of the expression level

The size factor and library size were calculated using the “calcNormFactors” function in the R package “edgeR”^75^ from the read counts that were normalized to the read counts of each transcript to be at the length of 1000, where *X*_t_ is the read counts per 1000 bps, *R*_t_ is the read counts that were mapped, *L*_t_ is the length of the transcript length, *S*_t_ is the library size, *N*_t_ is the normalization factor of RLE normalization, and TPM_t_ is the transcripts per million (TPM).

1 $$X_{\rm{t}} = \frac{{R_{\rm{t}}}}{{L_{\rm{t}}}}10^3$$

2 $${{{\mathrm{TPM}}}}_{\rm{t}} = X_{\rm{t}}\frac{1}{{S_{\rm{t}}N_{\rm{t}}}}10^6$$

The expression level for each protein-coding gene was normalized using RLE normalization, where the raw read counts were normalized by the number of the total read counts to be 1,000,000 after the read counts were divided by the product of the library size and size factor. The mean value of the two replicates was used for further analysis.

### Identification of DEGs

DEGs were identified using the “DESeq” function in the R package “DESeq2”^76^ by performing a Wald significance test between the gene expression levels before and after TNF stimulation for each time point to calculate the adjusted *p* values and fold change in expression for each gene in control and siIκBα. The expression levels of the genes were normalized using the RLE normalization method. Genes with adjusted *p* values <0.05 and log2 fold change ≥0 were classified as upregulated DEGs, and adjusted *p* values <0.05, and log2 fold change ≤0 as downregulated DEGs.

### Clustering and subclustering of DEGs

The DEGs in control and siIκBα were clustered into groups by their *z*-score normalized expression level using Fuzzy *c*-means clustering from the “cmeans” function in the R package “e1071.” Used parameters are: centers = 5 for control and 3 for siIκBα (number of clusters), iter.max = 2 (maximum number of iterations), method = cmeans (clustering method), *m* = 1.3 (fuzzy partition matrix), and dist = Euclidean (similarity measurement). Furthermore, for each TNF-induced cluster (ERGs, IRGs, and DRGs), we calculated the *z*-score normalized fold change for both conditions and identified the mean time point of the maximum expression level. The *z*-score normalized fold change was extracted at the corresponding time points until it reached the time point in data just before the calculated mean time point of the maximum expression level of all genes in each cluster. The extracted *z*-score normalized fold change in the control and siIκBα were combined for each gene. The genes were subclustered into two groups in each cluster based on these values using Fuzzy *c*-means clustering. Used parameters are: centers = 2 (number of clusters), iter.max = 2 (maximum number of iterations), method = cmeans (clustering method), *m* = 1.3 (fuzzy partition matrix), and dist = Euclidean (similarity measurement). Motif analysis was performed using “findMotifsGenome” command in HOMER^77^ version 4.10.4 for each cluster in control and siIκBα.

### Sequence mapping of single-cell ATAC-seq data

Single-cell ATAC-seq datasets containing two or more cells and samples that were contaminated were filtered out. A total of 989 cells remained in the control and 953 cells in the siIκBα, which were used for further analysis. In addition, a dataset was prepared in which all cells of the three replicates were aggregated into one dataset for each time point in each condition. The single-cell and aggregated datasets were aligned using Bowtie^78^ version 2.3.4.1, to build version UCSC26/hg38 of the human genome. Alignments were performed using the option “-S -p 8 -m 1” where -S indicates that alignments will be printed in SAM format; -p 8 indicates that eight parallel search threads will be launched; and -m 1 indicates that all alignments for a particular read will be suppressed if more than one reportable alignment exists. Picard (http://broadinstitute.github.io/picard/) version 1.119 was used to remove duplicates that arose during sample preparation. Mitochondrial chromosomes were removed from the mapped data using samtools^79^ version 1.9. The “bamCoverage” function in deepTools^80^ version 2.4.3 was used to quantify the reads mapped to the genome using the option “–binSize 1 -e 200 -ignoreForNormalization chrX -p max” where –binSize 1 indicates that each genomic region mapped by the reads are normalized to a bin of 1; -e 200 indicates that the reads will be extended to a fragment size of 200; -ignoreForNormalization chrX indicates that chromosome X will be excluded for computing the normalization; and -p max indicates that eight processors are used for the calculation.

### Peak region identification

Peak calling was performed with MACS2 (https://pypi.org/project/MACS2/) version 2.1.2.1 using the option “-f BAM -g hs -q 0.05 –mfold 6 50” where -f BAM indicates the format of the input file; -g hs indicates the mappable human genome size; -q 0.05 indicates the cutoff to call significant regions is 0.05; and –mfold 6 50 indicates the lower and upper threshold of the height of the ATAC-seq peaks.

### Quantification of chromatin accessibility

The signal of peak regions was calculated using “multiBigwigSummary” function in deepTools^80^, using the option “–BED” where a BED file with promoter regions defined as ±500 bps TSS of protein-coding genes from version GRCh38/hg38 of the human genome was provided. To normalize each signal at each time point, reads per million (RPM) were calculated, where the raw signal was normalized by the number of the total signal to be 1,000,000 after the read counts were divided by the product of the library size and size factor. The size factor and library size to normalize the ATAC-seq signal for each protein-coding gene were calculated using the TMM normalization method from the “calcNormFactors” function in the R package “edgeR”^75^.

### Identification and clustering of TNF-induced peak regions

Using the identified peak regions, significantly induced peak regions were detected for each time point after stimulation using the “getDifferentialPeaks” command in HOMER^77^ and these regions were merged. Regions that were not induced after TMM normalization were filtered out. The remaining regions were clustered based on their time-course chromatin accessibility. The chromatin accessibility at each time point was classified into a group in which its *z*-score normalized chromatin accessibility was smaller than 0 (*z*-score < 0) and a group whose *z*-score normalized chromatin accessibility was more than 0 (*z*-score ≥ 0). For each cluster, motif analysis was performed using the “findMotifsGenome” command in HOMER^77^ and identified clusters which showed enrichment of κB sites. From this result, we extracted only the regions that included κB sites in each cluster. The aggregated single-cell chromatin accessibility at those regions in each condition was calculated and a paired *t* test from “t.test” function in the R package “stats” was performed to determine whether they show statistical significance. In addition, we calculated the single-cell chromatin accessibility at those regions in each condition and performed a 10-fold cross validation using a Bayesian generalized linear model from the “train” function in the R package “caret” to calculate the accuracy of the classification between the single cells of control and siIκBα in those regions.

### Mathematical modeling

Basal nuclear NFκB activity from the two independent immunostaining results was subtracted from all time points in control and siIκBα. For replicate 1, the fold change of nuclear NFκB activity in control and siIκBα were calculated. Then, the fold change in control and siIκBα were converted together using the “rescale” function in the R package “scales” to span a range of 2–100 to avoid assay specific reductions of the dynamic range. The fold change in nuclear NFκB activity of replicate 2 from the immunostaining result in control was converted to span a range of 2–100 to avoid assay-specific reductions in the dynamic range. Then we converted the immunostaining result in the siIκBα of replicate 2 using the same scale used for the control. Scaling methods were different between the two replicates because the time-course nuclear NFκB activity of replicate 2 in both conditions was consistently lower than the activity of replicate 1 in both conditions. While the maximum activity in control which appeared at the first peak was also the highest among both conditions in replicate 1, the maximum activity among both conditions in replicate 2 appeared at late time points in siIκBα. Thus, to standardize the maximum activity of replicates 1 and 2, activity of replicate 2 in control was scaled individually to avoid the scaled maximum activity (which is 100 in replicate 1) to appear at late time points in siIκBα but to appear at the first peak in control, similar to the scaled maximum activity of replicate 1. Each of the scaled nuclear NFκB activities was interpolated to every second using the “pchipfun” function in the R package “pracma.” These interpolated data for each replicate were used as the input of all ODE models, which were numerically solved using the “ode” function in the R package “deSolve.”

### The simple model

A simple model was used to reproduce the transcription regulating mechanism that considers only the nuclear NFκB activity and its target gene. Where, *k*_syn_ is the synthesis rate constant for the target gene, *k*_deg_ is the mRNA degradation rate constant for the target gene, *K*_*D*_ is the NFκB-regulation strength constant for the target gene, *h* is the Hill function exponent for the target gene, and *τ* is the time between nuclear NFκB and mature poly A^+^ mRNA production.

3 $$\frac{{{\rm{d}}\left[ {{{{\mathrm{mRNA}}}}\left( t \right)} \right]}}{{{\rm{d}}t}} = k_{{\rm{syn}}}\left( {\frac{{\left( {K_D\left[ {{{{\mathrm{NFkB}}}}\left( {t - \tau } \right)} \right]} \right)^h}}{{\left( {K_D\left[ {{{{\mathrm{NFkB}}}}\left( {t - \tau } \right)} \right]} \right)^h + 1}}} \right) - k_{{\rm{deg}}}\left[ {{{{\mathrm{mRNA}}}}\left( t \right)} \right]$$

The free parameters *k*_deg_, *K*_*D*_, and *τ* were optimized with bound constraints (${{{\mathrm{2e - 5}}}} \,<\, k_{{\rm{deg}}} \,< \,{{{\mathrm{2e - 3}}}}$, ${{{\mathrm{0}}}}{{{\mathrm{.001}}}} < K_D < {{{\mathrm{1000}}}}$, and ${{{\mathrm{0}}}} < \tau < {{{\mathrm{7200}}}}$ sec). For simplicity, *h* and *k*_syn_ were fixed at 1.

### The IFFL model

The IFFL model^29^ was used to enable a transcription-regulating mechanism that is fold change detectable. This model considered the competitor that binds to the target gene promoter to interfere with the transcriptional regulation by NFκB, where, *k*_degTF_ is the mRNA degradation rate constant for the competitor TF gene, *K*_*D*TF_ is the NFκB-regulation strength constant for the competitor TF gene, *K*_*D*1_ is the NFκB-regulation strength constant for the target gene, *K*_*D*2_ is the competitor TF-regulation strength constant for the target gene, *h*_TF_ is the Hill function exponent for the competitor TF gene, and *τ*_TF_ is the time between nuclear TF and mature poly A^+^ mRNA production.

4 $$\frac{{{\rm{d}}\left[ {{{{\mathrm{TF}}}}\left( t \right)} \right]}}{{{\rm{d}}t}} = \left( {\frac{{\left( {K_{D{\rm{TF}}}\left[ {{{{\mathrm{NFkB}}}}\left( {t - \tau } \right)} \right]} \right)^{h_{{\rm{TF}}}}}}{{\left( {K_{D{\rm{TF}}}\left[ {{{{\mathrm{NFkB}}}}\left( {t - \tau } \right)} \right]} \right)^{h_{{\rm{TF}}}} + 1}}} \right) - k_{{\rm{degTF}}}\left[ {{{{\mathrm{TF}}}}\left( t \right)} \right]$$

5 $$\begin{array}{ll}\frac{{{\rm{d}}\left[ {{{{\mathrm{mRNA}}}}\left( t \right)} \right]}}{{{\rm{d}}t}}\,\, = & k_{{\rm{syn}}}\left( {\frac{{\left( {K_{D1}\left[ {{{{\mathrm{NFkB}}}}\left( {t - \tau } \right)} \right]} \right)^h}}{{\left( {K_{D1}\left[ {{{{\mathrm{NFkB}}}}\left( {t - \tau } \right)} \right]} \right)^h + \left( {K_{D2}\left[ {{{{\mathrm{TF}}}}\left( {t - \tau _{{\rm{TF}}}} \right)} \right]} \right)^h + 1}}} \right)\\& -\, k_{{\rm{deg}}}\left[ {{{{\mathrm{mRNA}}}}\left( t \right)} \right]\end{array}$$

The free parameters *k*_deg_, *K*_*D*1_, *K*_*D*2_, and *τ* were optimized with bound constraints (${{{\mathrm{2e - 5}}}} \,<\, k_{{\rm{deg}}}\, <\, {{{\mathrm{2e - 3}}}}$, ${{{\mathrm{0}}}}{{{\mathrm{.001}}}} \,<\, K_{D1},K_{D2} \,<\, {{{\mathrm{1000}}}}$, and ${{{\mathrm{0}}}} \,<\, \tau \,<\, {{{\mathrm{7200}}}}$ sec). For simplicity, *h* was fixed at 2, *h*_TF_ was fixed at 1, *k*_degTF_ was fixed at 8.022537e-6, *K*_*D*TF_ was fixed at 100, *τ*_TF_ was fixed at 7200 sec (refs ^36,44^), and *k*_syn_ was fixed at 1.

### The 3-state cycle model

The 3-state cycle model was constructed to recapitulate the promoter state transition from the active state (state *A*) to the closed state (state *C*), which induces transcriptional repression of the target gene. Where *K*_*D*3_ is the cooperativity of the active chromatin state at the promoter region of the target gene, and *k*_1_, *k*_2,_ *k*_*−*1_, *k*_−2_, and *k*_−3_ are the reaction rate constants.

6 $$\frac{{{\rm{d}}C}}{{{\rm{d}}t}} = - k_1\left( {\frac{{K_{D1}\left[ {{{{\mathrm{NFkB}}}}\left( t \right)} \right]}}{{K_{D1}\left[ {{{{\mathrm{NFkB}}}}\left( t \right)} \right] + 1}}} \right) \cdot C + k_{ - 1} \cdot O + k_{ - 3} \left( {\frac{{K_{D3} \cdot A}}{{K_{D3} \cdot A + 1}}} \right)$$

7 $$\begin{array}{ll}\frac{{{\rm{d}}O}}{{{\rm{d}}t}} = k_1\left( {\frac{{K_{D1}\left[ {{{{\mathrm{NFkB}}}}\left( t \right)} \right]}}{{K_{D1}\left[ {{{{\mathrm{NFkB}}}}\left( t \right)} \right] + 1}}} \right) \cdot C - k_2\left( {\frac{{K_{D2}\left[ {{{{\mathrm{NFkB}}}}\left( t \right)} \right]}}{{K_{D2}\left[ {{{{\mathrm{NFkB}}}}\left( t \right)} \right] + 1}}} \right)\\ \qquad\,\,\, \cdot O - k_{ - 1} \cdot O + k_{ - 2} \cdot A\end{array}$$

8 $$\frac{{{\rm{d}}A}}{{{\rm{d}}t}} = k_2\left( {\frac{{K_{D2}\left[ {{{{\mathrm{NFkB}}}}\left( t \right)} \right]}}{{K_{D2}\left[ {{{{\mathrm{NFkB}}}}\left( t \right)} \right] + 1}}} \right) \cdot O - k_{ - 2} \cdot A - k_{ - 3} \left( {\frac{{K_{D3} \cdot A}}{{K_{D3} \cdot A + 1}}} \right)$$

9 $$1 = C + O + A$$

10 $$\frac{{{\rm{d}}\left[ {{{{\mathrm{mRNA}}}}\left( t \right)} \right]}}{{{\rm{d}}t}} = k_{{\rm{syn}}} \cdot A\left( {t - \tau } \right) - k_{{\rm{deg}}}\left[ {{{{\mathrm{mRNA}}}}\left( t \right)} \right]$$

The free parameters *k*_1_, *k*_2_, *k*_deg_, *K*_*D*1_, *K*_*D*2_, and *τ* were optimized with bound constraints ($2{\rm{e}} - 5 \,< \, k_{{\rm{deg}}} \,<\, 2{\rm{e}} - 3$, $6{\rm{e}}-5 \, < \, k_1 \, < \, 6{\rm{e}}-3, \, 0.007 \, < \, k_2 \, < \, 69.31$, $0.001 \, < \, K_{D1},K_{D2} \, < \, 1000$, and $0 \, < \, \tau \, < \, 7200$ sec). For simplicity, *k*_−1_ was fixed at 0.01 × *k*_1_, *k*_−2_ was fixed at 0.01 × *k*_2_, *k*_−3_ was fixed at 1, *K*_*D*3_ was fixed at 0.5, and *k*_syn_ was fixed at 1.

### The model v4

Model v4 is a combination of the 3-state cycle and the IFFL models, where *K*_*D*TF2_ is the competitor TF regulation strength constant for the target gene.

11 $$\frac{{{\rm{d}}C}}{{{\rm{d}}t}} = - k_1\left( {\frac{{K_{D1}\left[ {{{{\mathrm{NFkB}}}}\left( {t - \tau } \right)} \right]}}{{K_{D1}\left[ {{{{\mathrm{NFkB}}}}\left( {t - \tau } \right)} \right] + 1}}} \right) \cdot C + k_{ - 1} \cdot O + k_{ - 3}\left( {\frac{{K_{D3} \cdot A}}{{K_{D3} \cdot A + 1}}} \right)$$

12 $$\begin{array}{ll}\frac{{{\rm{d}}O}}{{{\rm{d}}t}} \,\,=& k_1\left( {\frac{{K_{D1}\left[ {{{{\mathrm{NFkB}}}}\left( {t - \tau } \right)} \right]}}{{K_{D1}\left[ {{{{\mathrm{NFkB}}}}\left( {t - \tau } \right)} \right] + 1}}} \right) \cdot C - k_2\left( {\frac{{K_{D2}\left[ {{{{\mathrm{NFkB}}}}\left( {t - \tau } \right)} \right]}}{{K_{D2}\left[ {{{{\mathrm{NFkB}}}}\left( {t - \tau } \right)} \right] + 1}}} \right)\\& \cdot O - k_{ - 1} \cdot O + k_{ - 2} \cdot A\end{array}$$

13 $$\frac{{{\rm{d}}A}}{{{\rm{d}}t}} = k_2\left( {\frac{{K_{D2}\left[ {{{{\mathrm{NFkB}}}}\left( {t - \tau } \right)} \right]}}{{K_{D2}\left[ {{{{\mathrm{NFkB}}}}\left( {t - \tau } \right)} \right] + 1}}} \right) \cdot O - k_{ - 2} \cdot A - k_{ - 3}\left( {\frac{{K_{D3} \cdot A}}{{K_{D3} \cdot A + 1}}} \right)$$

14 $$\frac{{{\rm{d}}\left[ {{{{\mathrm{TF}}}}\left( t \right)} \right]}}{{{\rm{d}}t}} = \left( {\frac{{\left( {K_{D{\rm{TF}}}\left[ {{{{\mathrm{NFkB}}}}\left( t \right)} \right]} \right)^{h_{{\rm{TF}}}}}}{{\left( {K_{D{\rm{TF}}}\left[ {{{{\mathrm{NFkB}}}}\left( t \right)} \right]} \right)^{h_{{\rm{TF}}}} + 1}}} \right) - k_{{\rm{degTF}}}\left[ {{{{\mathrm{TF}}}}\left( t \right)} \right]$$

15 $$1 = C + O + A$$

16 $$\begin{array}{ll}\frac{{{\rm{d}}\left[ {{{{\mathrm{mRNA}}}}\left( t \right)} \right]}}{{{\rm{d}}t}}\,\, =& k_{{\rm{syn}}}\left( {\frac{{\left( {A\left( {t - \tau } \right)} \right)^h}}{{\left( {A\left( {t - \tau } \right)} \right)^h + \left( {K_{D{\rm{TF}}2}\left[ {{{{\mathrm{TF}}}}\left( {t - \tau _{{\rm{TF}}}} \right)} \right]} \right)^h + 1}}} \right)\\& -\, k_{{\rm{deg}}}\left[ {{{{\mathrm{mRNA}}}}\left( t \right)} \right]\end{array}$$

The free parameters *k*_deg_, *k*_1_, *k*_2_, *K*_*D*1_, *K*_*D*2_, *K*_*D*TF2_, and *τ* were optimized with bound constraints ($2{\rm{e}} - 5 \,<\, k_{{\rm{deg}}} \,<\, 2{\rm{e}} - 3$, $6{\rm{e}}-5 \, < \, k_1 \, < \, 6{\rm{e}}-3, \, 0.007 \, < \, k_2 \,< \, 69.315$, $0.001 \, < \, K_{D1},K_{D2},K_{DTF2} \, < \, 1000$, and $0 \,< \, \tau \,<\, 7200$ sec). For simplicity, *h* was fixed at 2, *h*_TF_ was fixed at 1, *k*_degTF_ was fixed at 8.022537e−6, *K*_*D*TF_ was fixed at 100, *τ*_TF_ was fixed at 7200 sec, *k*_−1_ was fixed at 0.01 × *k*_1_, *k*_−2_ was fixed at 0.01 × *k*_2_, *k*_−3_ was fixed at 1, *K*_*D*3_ was fixed at 0.5, and *k*_syn_ was fixed at 1.

### Model maturation

Parameters for each model were optimized using the subplex algorithm from the “sbplx” function in the R package “nloptr” to minimize the normalized RMSD (which was calculated using the “rmsd” function in the R package “bio3d”^81^) between max-normalized measured data and max-normalized simulated results that span from 0 to 1 and the difference between the simulated expression at the start of steady state (−60 min) and the end of steady state (0 min). RMSD is calculated by the following formula, where *x*_*i*_ is the expected value, *y*_*i*_ are the observed values, and *n* is the total number of values. Max-normalized expression of measured data was calculated after subtracting the basal expression from the expression for all time points in each condition.

17 $${{{\mathrm{RMSD}}}} = \sqrt {\frac{1}{n}\mathop {\sum}\limits_{i = 1}^n {\left\| {x_i - y_i} \right\|^2} }$$

### Parameter optimization process and identification of concordant parameter sets

To identify the best-fit parameter, a two-step parameter optimization process was applied. First, using the scaled input nuclear NFκB activity, we simulated the expression and calculated the time-course fold change in the expression in control and siIκBα. The time-course fold change in expression from the entire dataset was also calculated, and these were max-normalized to span a range of 0 to1 for both simulation and data. Next, we minimized the nRMSD between the max-normalized fold change between the simulation and data in the control for 100 initial parameter sets. Within these optimized parameter sets, we selected parameter sets with nRMSD <0.5, some of which were set to 0.6 or 0.7, and identified the minimum and maximum values for each parameter. Using these two values as the lower and upper bound constraints, we further produced 100 initial parameter sets and optimized them by minimizing the nRMSD of the max-normalized fold change between the simulation and data in siIκBα.

After performing this optimization process for two biological replicates, we calculated the score for each optimized parameter set for each biological replicate. We identified the concordant optimized parameter sets between replicates 1 and 2 to ensure the robustness of each parameter value. First, we generated all possible pairs of optimized parameter sets from replicates 1 and 2 (100 optimized parameter sets in replicate 1 × 100 optimized parameter sets in replicate 2 = 10,000 pairs). For the score of parameters *k*_deg_ and *K*_*D*_, the larger parameter value in either replicate 1 or replicate 2 was divided by the smaller parameter value in either replicate 1 or 2. For the score of *τ*, the absolute value of the difference between *τ* in replicate 1 and *τ* in replicate 2 was calculated. The nRMSD score was the sum of the nRMSD in replicate 1 and nRMSD in replicate 2 for the ERGs, IRGs, and DRGs from the simple and IFFL models. The method to identify the concordant parameter sets for the ERGs in subcluster 2 is different for the 3-state cycle model and model v4, where we only calculated the score for *k*_1_, *k*_2_, *k*_deg_, *τ*, and nRMSD. The method to identify concordant parameter sets for IRGs and DRGs from 3-state cycle model and model v4 are the same as simple model and IFFL model. The total score of these scores was identified for each optimized parameter pair, and the parameter pair with the smallest total score for each gene was identified.

### Chromatin remodeling at promoter regions of post-induction repressed ERGs

There were 11 post-induction repressed genes that were best demonstrated by the 3-state cycle model or model v4. These genes all belonged to the ERGs in subcluster 2, and all the other genes that were best demonstrated by any of the four models were 89 in total. The peak regions at each time point in control and siIκBα that were included in the promoter regions (±500 bps TSS) of these 100 genes were identified. For these peak regions, motif analysis was performed using the “findMotifsGenome” command in HOMER^77^ to identify peak regions which showed enrichment of κB sites. From this result, only the peak regions that included κB sites were further extracted. When κB site enriched regions were identified for multiple time points, regions were merged using the “mergePeaks” command in HOMER^77^ using the default setting.

The signal of these peak regions was calculated using “multiBigwigSummary” function in deepTools^80^, using the option “--BED” where a BED file with promoter regions defined as ±500 bps TSS of protein-coding genes from version GRCh38/hg38 of the human genome was provided. To normalize each signal at each time point, RPM were calculated, where the raw signal was normalized by the number of the total signal to be 1,000,000 after the read counts were divided by the product of the library size and size factor. The size factor and library size to normalize the ATAC-seq signal for each protein-coding gene were calculated using the TMM normalization method from the “calcNormFactors” function in the R package “edgeR”^75^. The κB site-enriched regions that showed a start of decrease in chromatin accessibility at least at 30 or 75 min in both Ctrl and siIκBα were selected and *z*-score normalized for each condition for heatmaps. The heatmaps of the time-course fold change in expression of the 11 post-induction repressed ERGs in Ctrl and siIκBα were max-normalized from 0 to 1 for each condition using the gene expression data.

## Supplementary information

Supplementary Information

## Data Availability

All sequence data reported in this paper have been deposited in the DNA Data Bank of Japan with accession numbers DRA011742 and DRA011743 that are publicly accessible at https://www.ddbj.nig.ac.jp/index-e.html.

## Code availability

The source code necessary for reproducing figures, model fitting, and simulation are available on the GitHub website: https://github.com/okadalabipr/Ando2021.git.
