# Author Correction: IκBα is required for full transcriptional induction of some NFκB-regulated genes in response to TNF in MCF-7 cells

**DOI:** 10.1038/s41540-022-00239-4

**Published:** 2022-08-04

**Authors:** Minami Ando, Shigeyuki Magi, Masahide Seki, Yutaka Suzuki, Takeya Kasukawa, Diane Lefaudeux, Alexander Hoffmann, Mariko Okada

**Affiliations:** 1 Institute for Protein Research, Osaka University, 3-2 Yamadaoka, Suita, Osaka 565-0871 Japan; 2 Department of Computational Biology and Medical Sciences, Graduate School of Frontier Sciences, The University of Tokyo, Kashiwa, Chiba 277-8568 Japan; 3 RIKEN Center for Integrative Medical Sciences, Yokohama, Kanagawa 230-0045 Japan; 4 UCLA Department of Microbiology, Immunology and Molecular Genetics, 570 Boyer Hall, Los Angeles, CA 90095 USA; 5 Center for Drug Design and Research, National Institutes of Biomedical Innovation, Health and Nutrition, Ibaraki, Osaka 567-0085 Japan; 6 Institute for Chemical Research, Kyoto University, Kyoto, 611-0011 Japan

**Keywords:** Regulatory networks, Molecular biology

Correction to: *npj Systems Biology and Applications* 10.1038/s41540-021-00204-7, published online 01 December 2021

In the original version of this Article, a number of typos were present in several sections of the Methods: The IFFL model, The 3-state cycle model, and The model v4. In particular, units were clarified for τ, correct values were given for constants *h*, *h*_TF_, *k*_1_, *k*_2_, *K*_*D*3_, *K*_*DTF*2_, and equations 6 and 8 were corrected. The correction have been performed in both the HTML and PDF versions of the article.

