# Supplementary material for: IκBα is required for full transcriptional induction of some NFκB-regulated genes in response to TNF in MCF-7 cells
Source: NPJ Syst Biol Appl. 2021 Dec 1;7:42. doi: 10.1038/s41540-021-00204-7 (PMC8636565; doi:10.1038/s41540-021-00204-7)
Supplement: Supplementary file 1 — Supplementary Information [file 41540_2021_204_MOESM1_ESM.pdf]

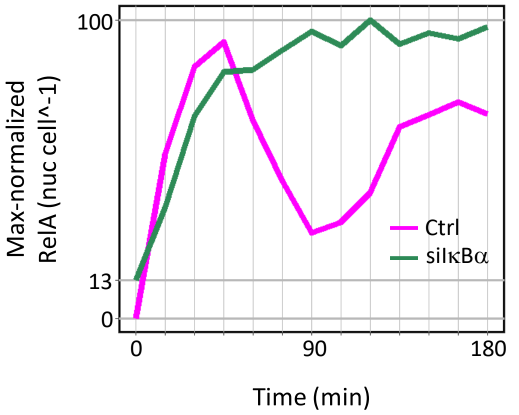

Supplementary Figure 1. Difference in the basal level of nuclear NFκB abundance between presence and absence of IκBα
Time course nuclear NFκB abundance of two biological replicates were max-normalized together from 0 to 100.

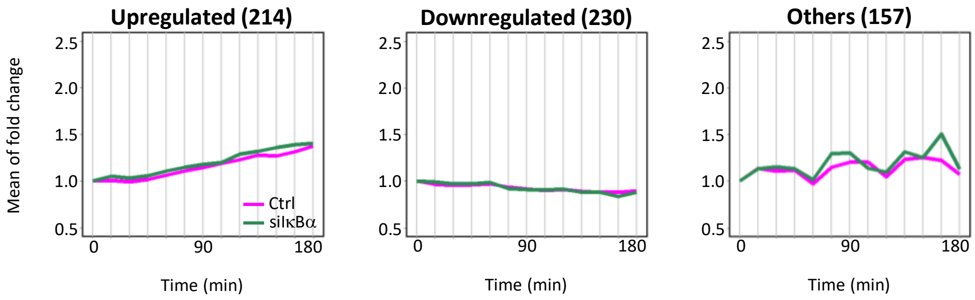

Supplementary Figure 2. Three clusters of DEGs that were unique to siIκBα (IκBα knockdown)
601 DEGs that were unique to siIκBα were classified into upregulated, downregulated and others clusters. Line graphs show the mean of time course fold change in expression.

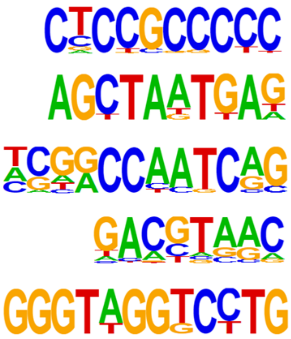

Rank		P-value	Target %	Motif
1		1e-11	29.23 %	Sp5
2		1e-10	3.59 %	LXH9
3		1e-9	8.21 %	NFYB
4		1e-9	27.18 %	GMEB2
5		1e-9	2.56 %	ZNF467

Supplementary Figure 3. Motif analysis results at promoter regions of upregulated DEGs (Differentially Expression Genes) that were unique to siIκBα (IκBα knockdown) cells

Motif analysis results at the promoter regions of the upregulated DEGs that were unique to siIκBα condition.

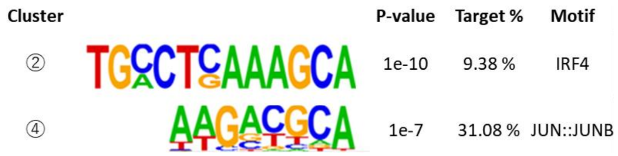

Supplementary Figure 4. Motif analysis results of other inflammatory transcription factors at TNF (Tumor Necrosis Factor)-induced κB site enriched clusters in Ctrl (Control)

Motif analysis results showed that AP-1 and IRF4 binding sites were also enriched at κB site enriched clusters in Ctrl.

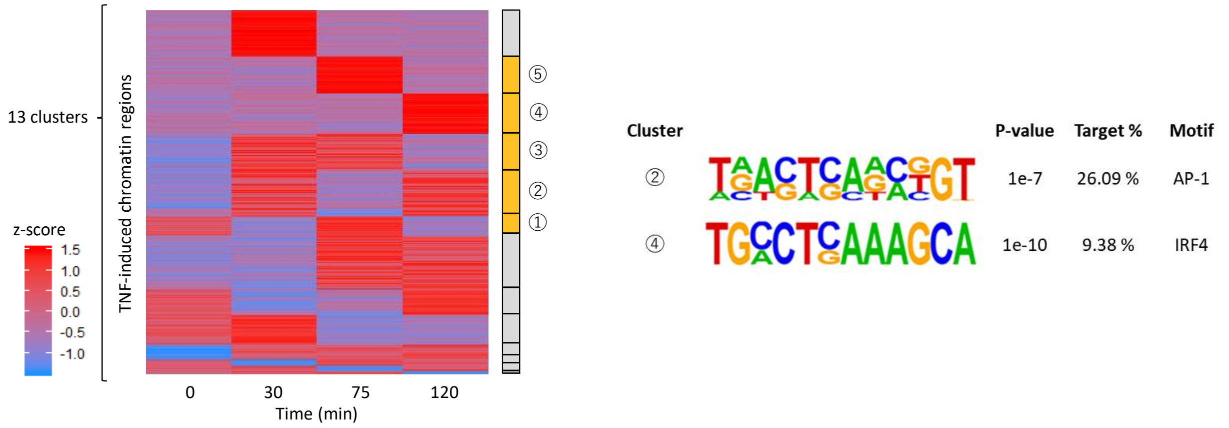

Supplementary Figure 5. TNF (Tumor Necrosis Factor)-induced chromatin region clusters and motif analysis results of other inflammatory transcription factors at κB site enriched clusters in siIκBα (IκBα knockdown)

Classified TNF-induced regions in siIκBα into 13 time course clusters and identified 5 clusters which were enriched with κB sites. Chromatin accessibility was normalized to z-score, where red shows high chromatin accessibility and blue shows low chromatin accessibility in the heatmap. In addition to the enrichment of κB sites, these clusters also showed enrichment of AP-1 and IRF4 binding sites.

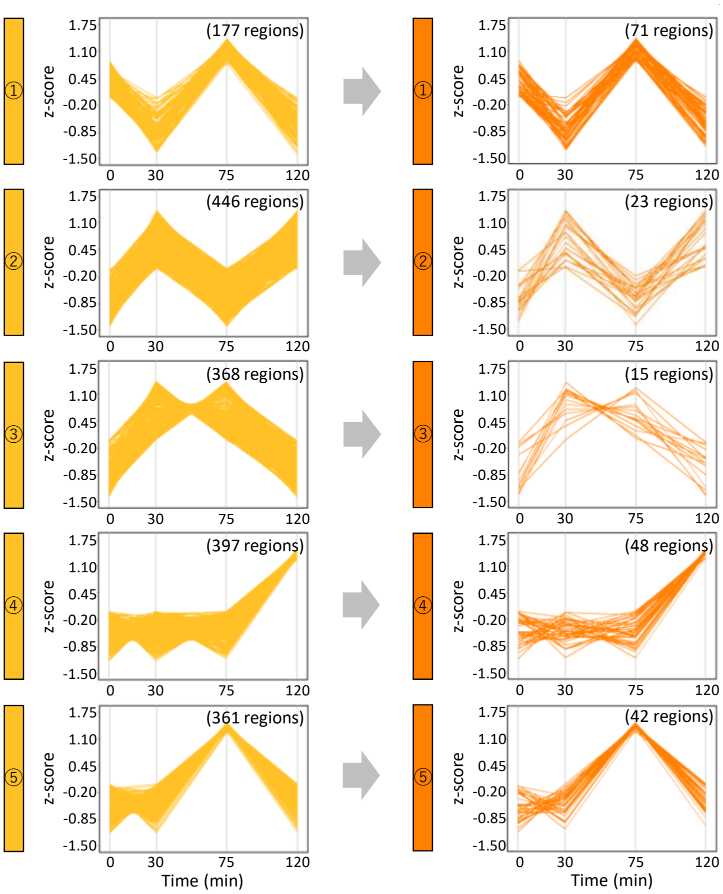

Supplementary Figure 6. Extraction of κB site detected regions from κB site enriched clusters in siIκBα (IκBα knockdown)
Clusters enriched with κB sites and extracted κB site detected regions in siIκBα. Time course chromatin accessibility was z-score normalized for each κB site enriched region.

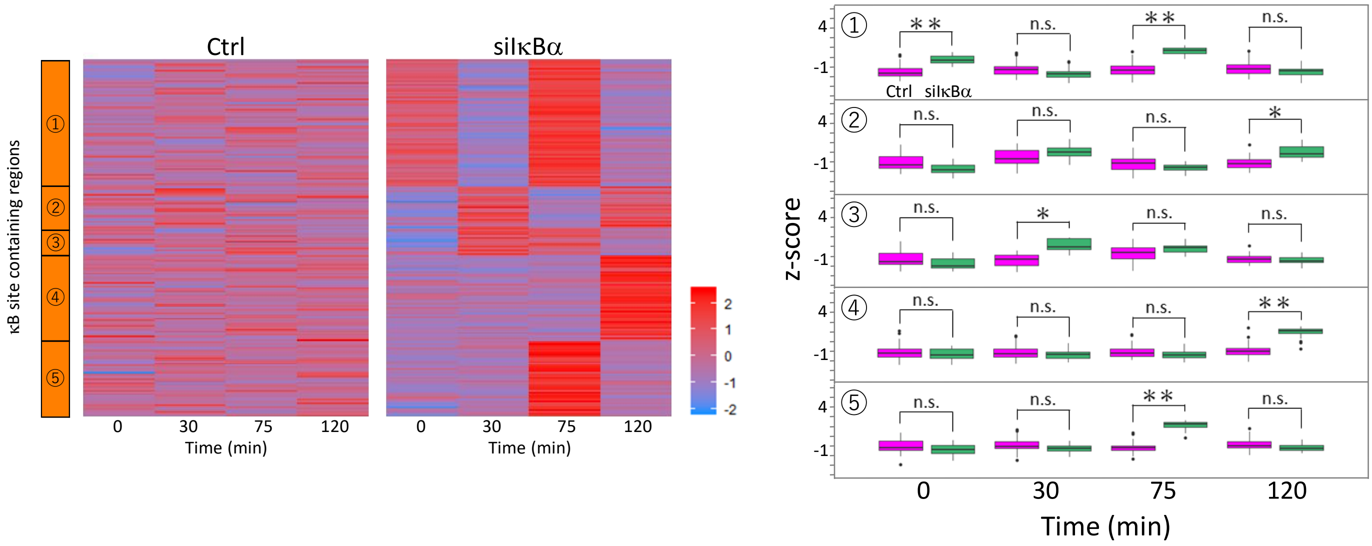

Supplementary Figure 7. Comparison of chromatin accessibility between presence and absence of IκBα at κB site detected regions in siIκBα (IκBα knockdown)

Time course chromatin accessibility in Ctrl (Control) and siIκBα at κB site detected regions in siIκBα, and comparison of time course chromatin accessibility between two conditions at κB site-enriched regions in siIκBα. Chromatin accessibility was normalized to z-score, where red shows high chromatin accessibility and blue shows low chromatin accessibility in the heatmap. Statistical tests were performed for chromatin accessibility between Ctrl and siIκBα at these regions in Ctrl and siIκBα (*: p-value < 0.01, **: p-value < 0.0001 and n.s.: p-value ≥ 0.01 by one-tailed Wilcoxon rank sum test). The center line indicates the median, the upper and lower hinges indicate the first and third quartiles, the upper whisker extends from the hinge to the largest value no further than 1.5 × IQR (interquartile range) from the hinge, the lower whisker extends from the hinge to the smallest value at most 1.5 × IQR of the hinge, and the points indicate the outliers.

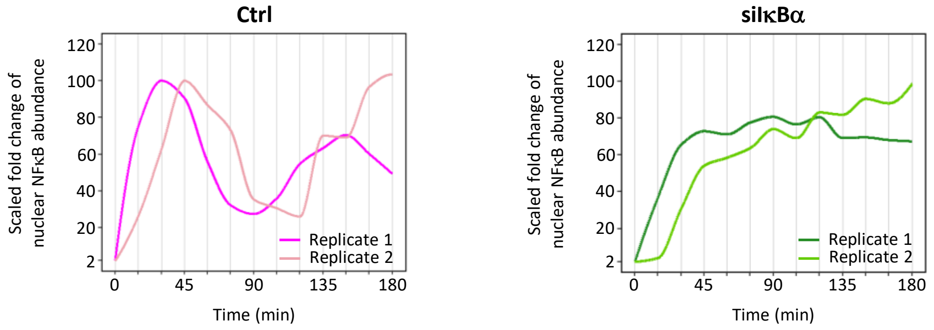

Supplementary Figure 8. The scaled fold change of nuclear NFκB abundance as the input data for mathematical modeling
For replicate 1, fold change for all time points in each condition were calculated. Fractional nuclear RelA abundance (nuc cell^-1) in Ctrl and siIκBα were normalized together to span a range of 2 – 100 (50-fold) to avoid assay specific reductions of the dynamic range. For replicate 2, after calculating the fold change for all time points in each condition, fractional nuclear RelA abundance (nuc cell^-1) in Ctrl was normalized to span a range of 2 – 100 to standardize the maximum activity with the scaled Ctrl data in replicate 1. Then, the measured data in siIκBα was normalized using the same scale used in Ctrl.

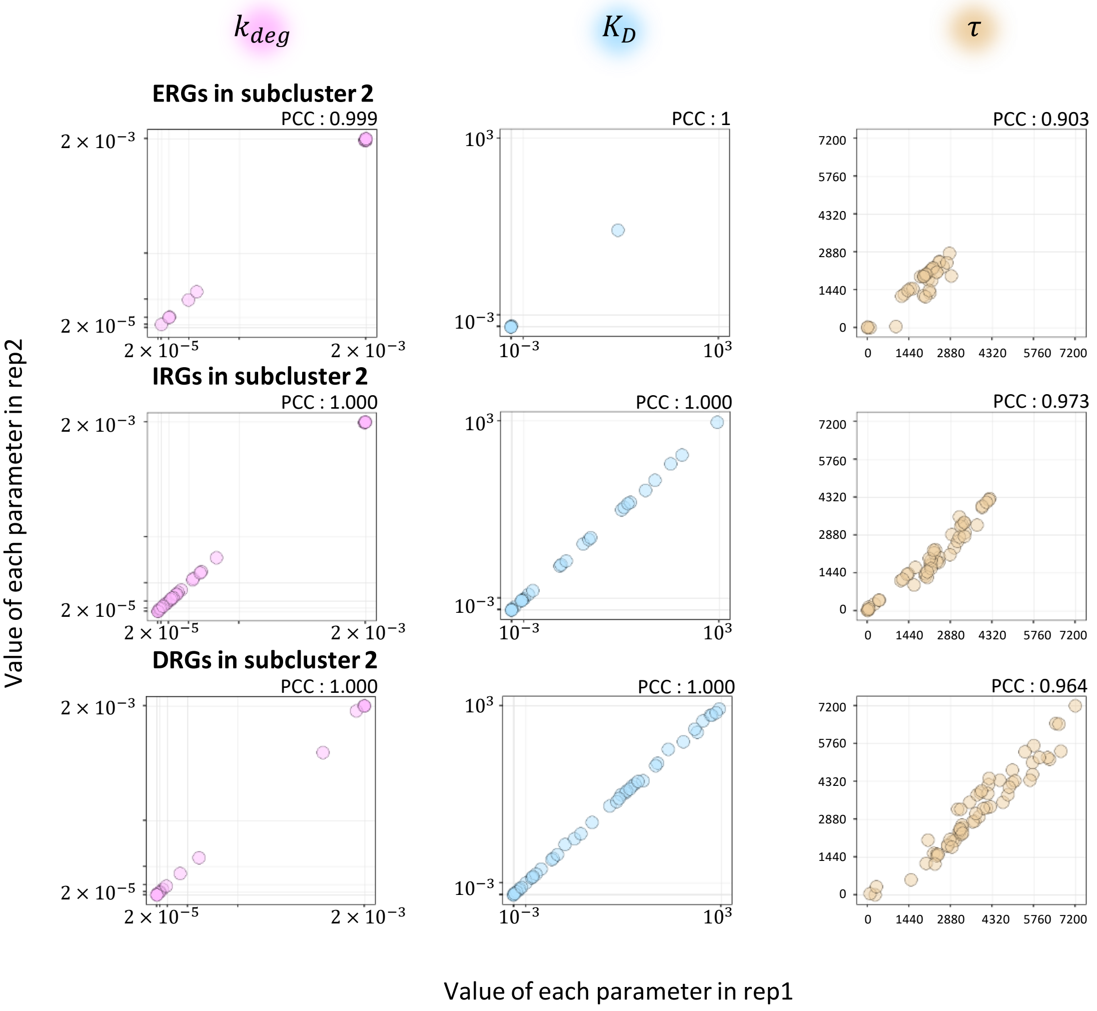

Supplementary Figure 9. PCC (Pearson Correlation Coefficient) of the values of each optimized parameter using the simple model

Optimized parameters from the concordant parameter set using the simple model of replicate 1 and replicate 2 for each gene are plotted in dots. They showed positive correlation between the 2 replicates.

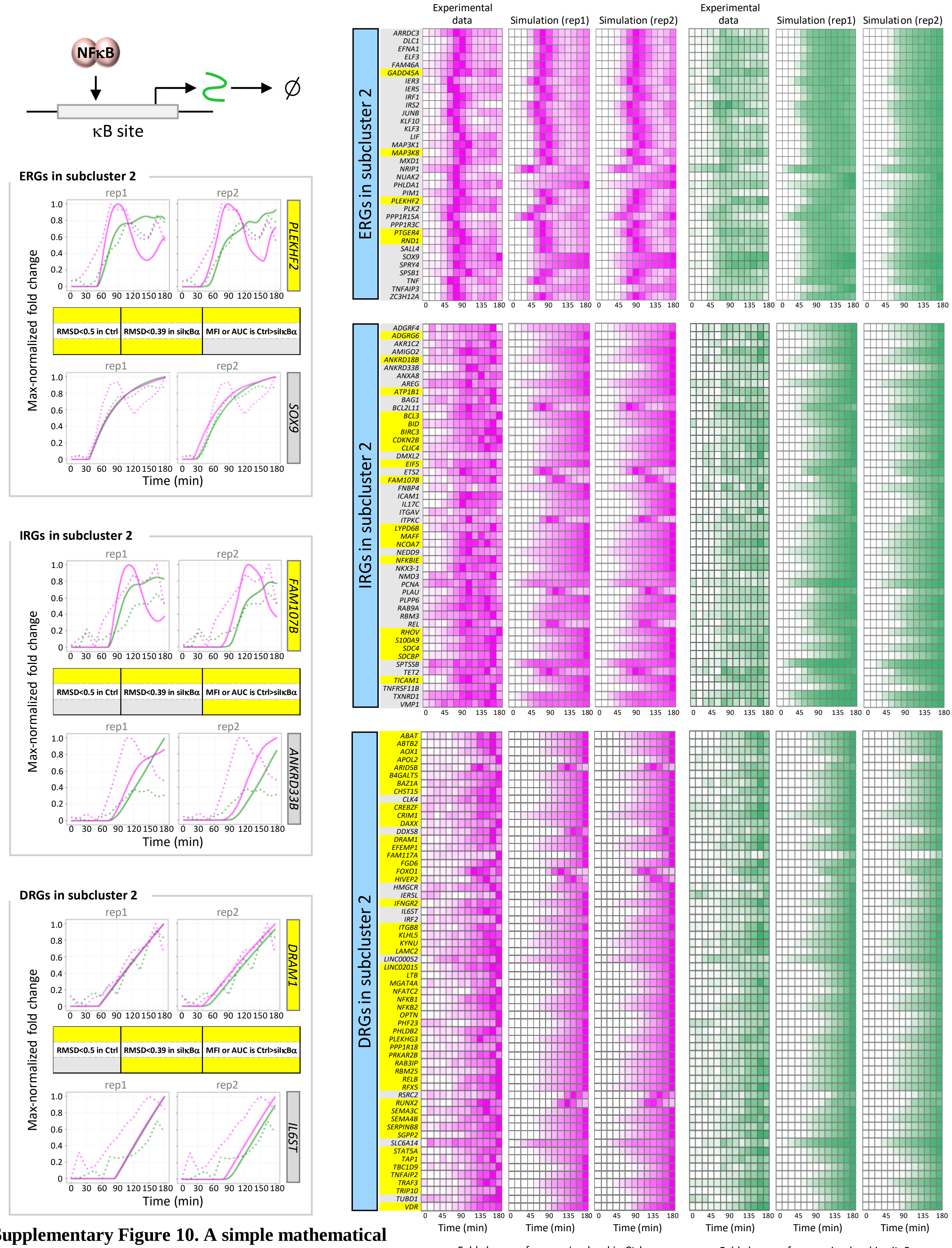

Supplementary Figure 10. A simple mathematical model recapitulates NFκB target gene activation but not post-induction repression or reduced expression

Schematic diagram of the simple mathematical model. Heatmaps of the time course gene expression from experimental results and data-fit from the simple model. Genes highlighted with yellow and grey indicate whether the model is acceptable or not (definition of acceptable genes: nRMSD in Ctrl > 0.5, nRMSD in siIκBα > 0.39 and AUC of fold change in expression should be Ctrl > siIκBα or max-fold induction should be Ctrl > siIκBα). Line graphs of representative genes from ERGs in subcluster 2, IRGs in subcluster 2 and DRGs in subcluster 2 showing good and bad fits between the simulation results and the experimental data. Yellow color bars show that simulation were acceptable for both replicates, and grey color bars show that simulation was not acceptable for at least either one of the replicates based on the definition.

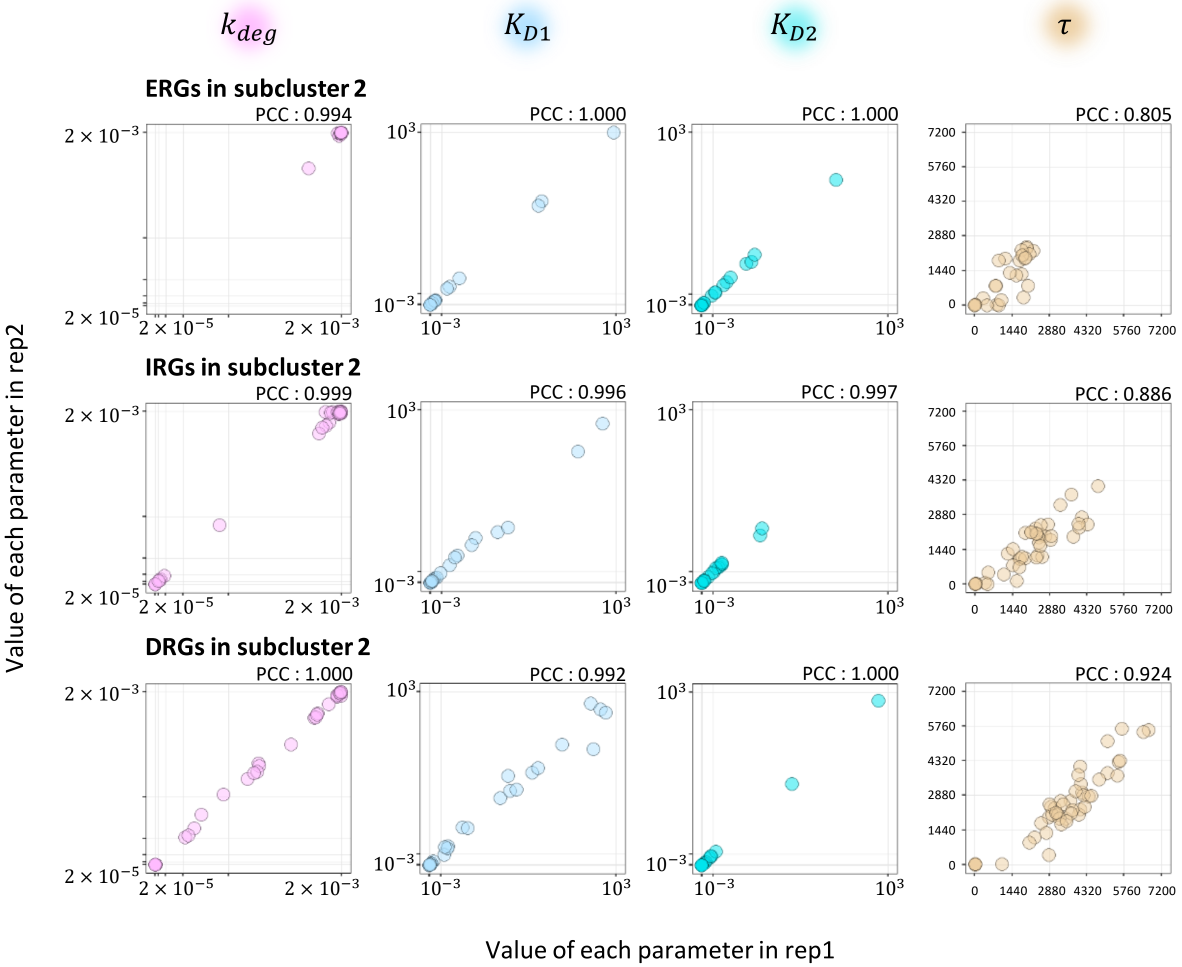

Supplementary Figure 11. PCC (Pearson Correlation Coefficient) of the values of each optimized parameter using the IFFL (incoherent feedforward loop) model

Optimized parameters from the concordant parameter set using the IFFL model of replicate 1 and replicate 2 for each gene are plotted in dots. They showed positive correlation between the 2 replicates.

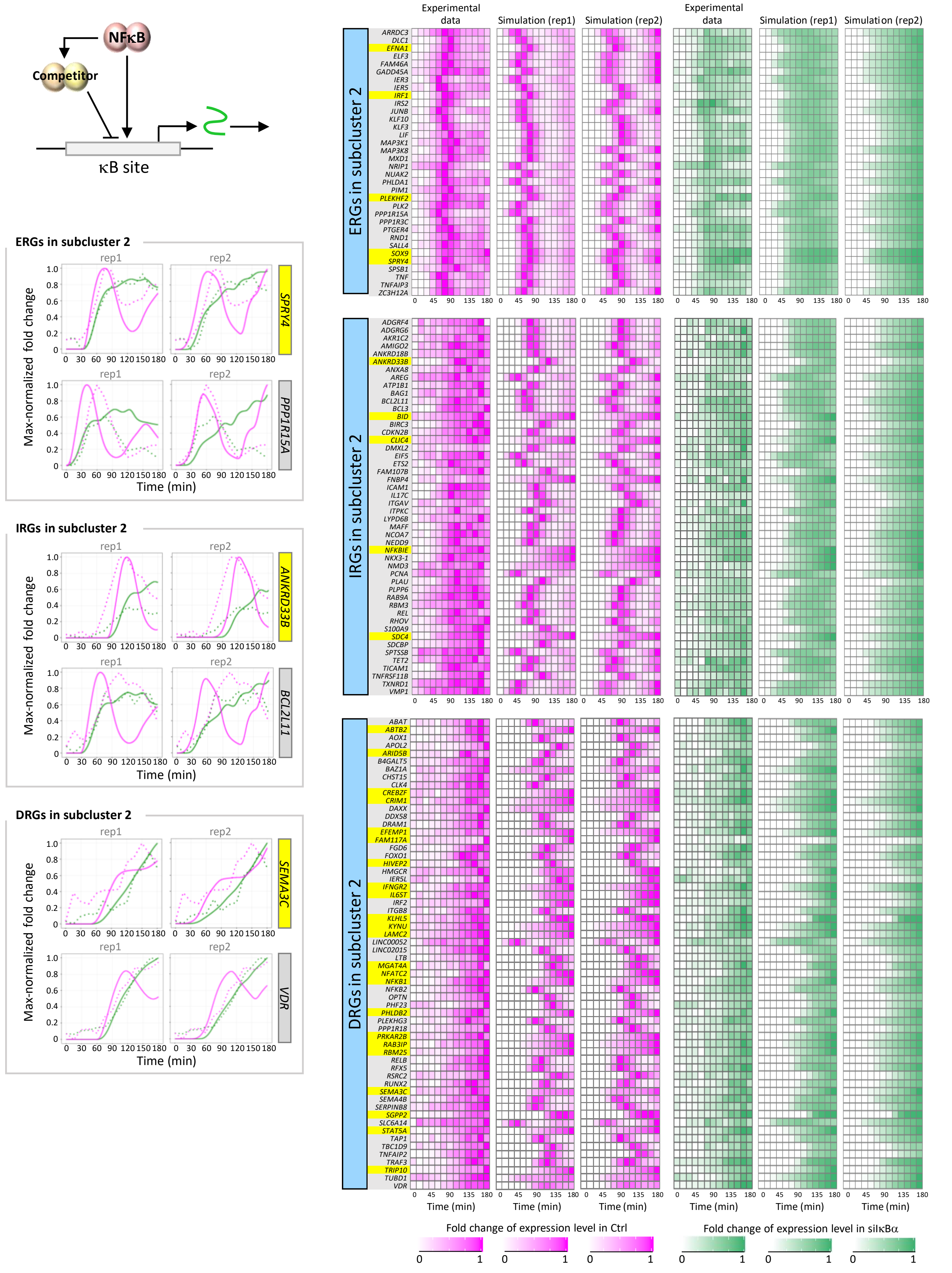

Supplementary Figure 12. An IFFL (incoherent feedforward loop) accounts for reduced gene expression
Schematic diagram of the IFFL model. Heatmaps of the time course gene expression from experimental results and data-fit from the IFFL model. Genes highlighted with yellow and grey indicate whether the model is acceptable or not (definition of acceptable genes: nRMSD in Ctrl > 0.5, nRMSD in siIκBα > 0.39 and AUC of fold change in expression should be Ctrl > siIκBα or max-fold induction should be Ctrl > siIκBα). Line graphs of representative genes from ERGs in subcluster 2, IRGs in subcluster 2 and DRGs in subcluster 2 showing good and bad fits between the simulation results and the experimental data.

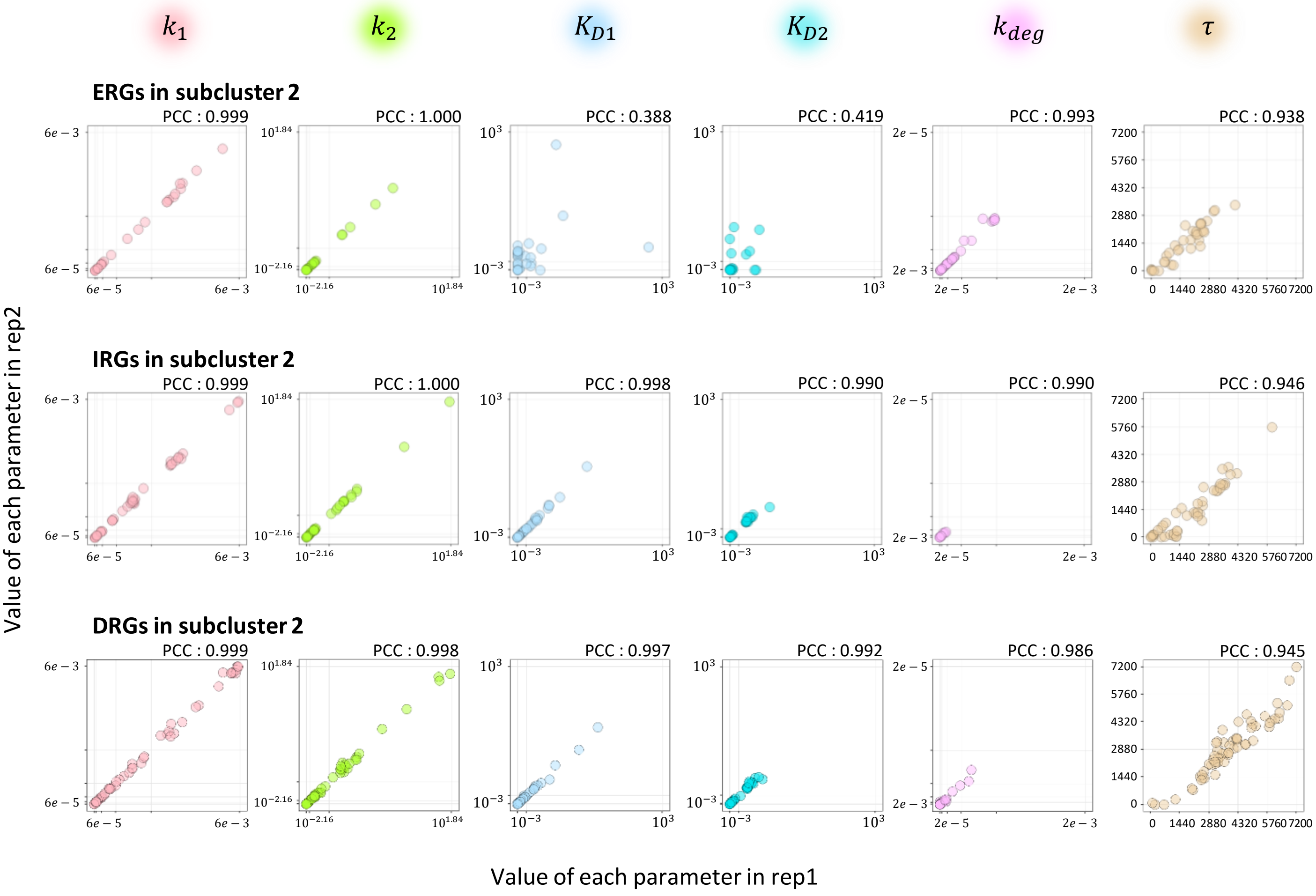

Supplementary Figure 13. PCC (Pearson Correlation Coefficient) of the values of each optimized parameter using the 3-state cycle model

Optimized parameters from the concordant parameter set using the 3-state cycle model of replicate 1 and replicate 2 for each gene are plotted in dots. They showed positive correlation between the 2 replicates.

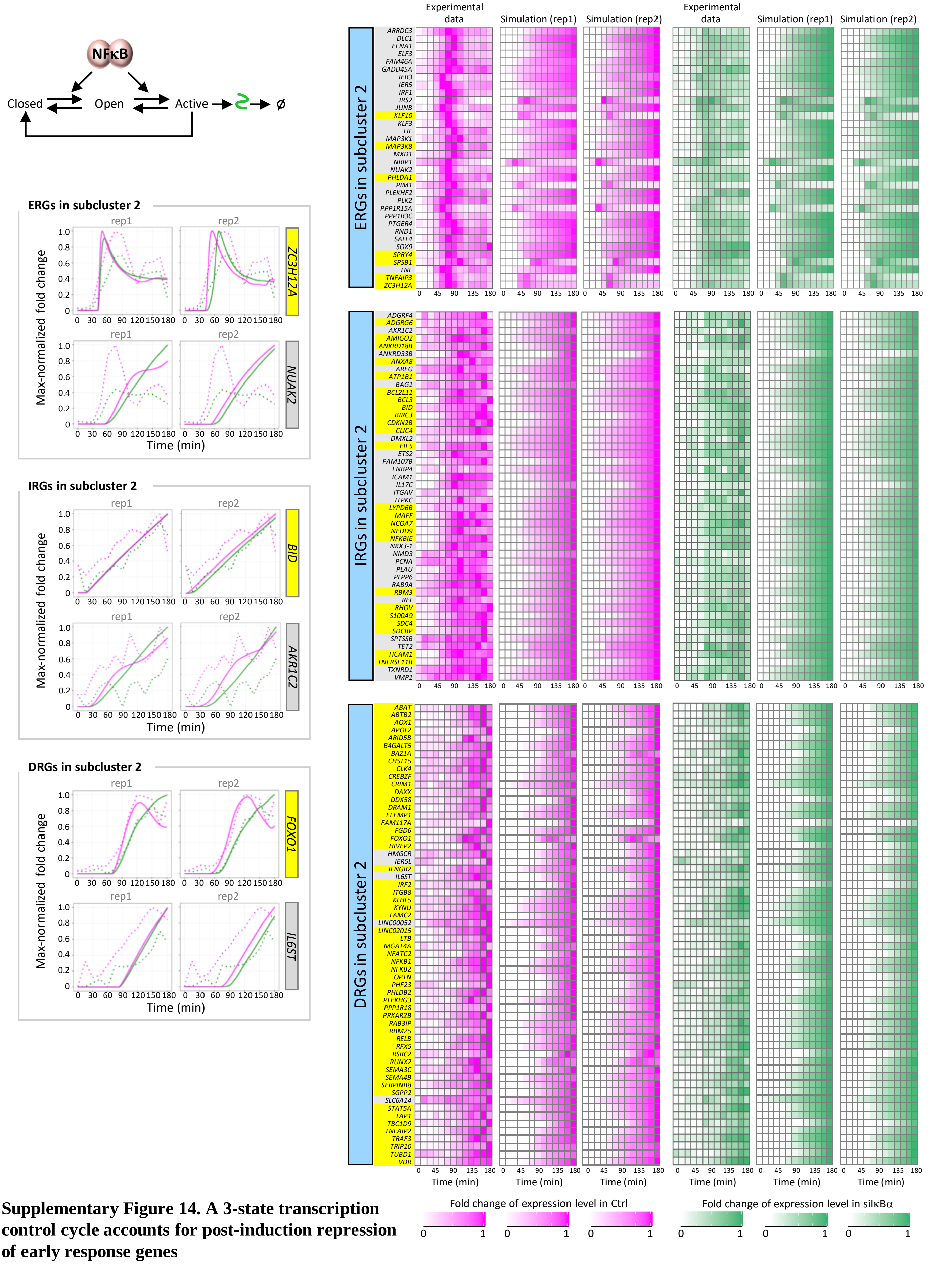

Supplementary Figure 14. A 3-state transcription control cycle accounts for post-induction repression of early response genes

Schematic diagram of the 3-state cycle model. Heatmaps of the time course gene expression from experimental results and data-fit from the 3-state transcription control cycle model. Genes highlighted with yellow and grey indicate whether the model is acceptable or not (definition of acceptable genes: nRMSD in Ctrl > 0.5, nRMSD in siIκBα > 0.39 and AUC of fold change in expression should be Ctrl > siIκBα or max-fold induction should be Ctrl > siIκBα). Line graphs of representative genes from ERGs in subcluster 2, IRGs in subcluster 2 and DRGs in subcluster 2 showing good and bad fits between the simulation results and the experimental data.

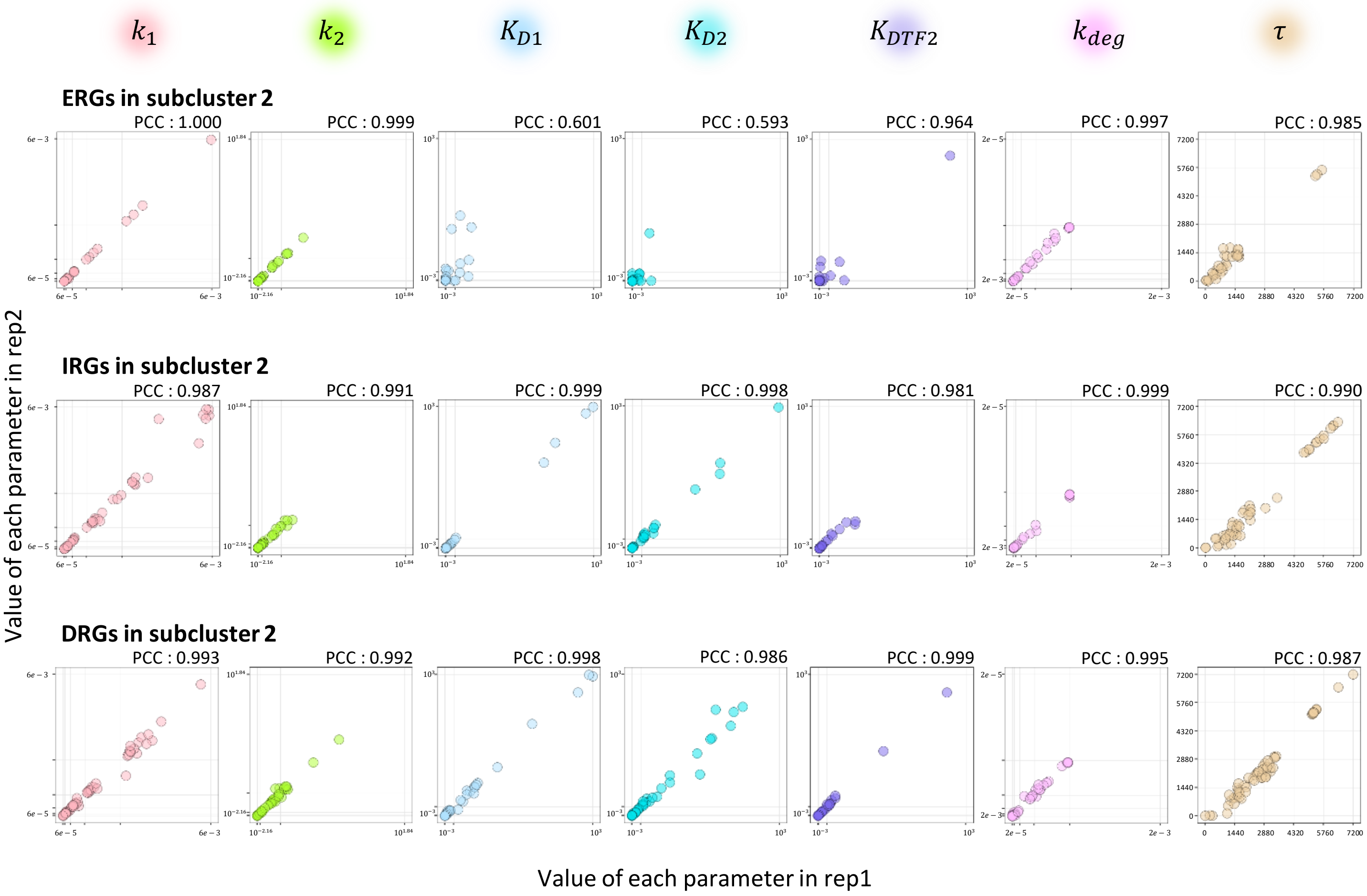

Supplementary Figure 15. PCC (Pearson Correlation Coefficient) of the values of each optimized parameter using model v4

Optimized parameters from the concordant parameter set using model v4 of replicate 1 and replicate 2 for each gene are plotted in dots. They showed positive correlation between the 2 replicates.

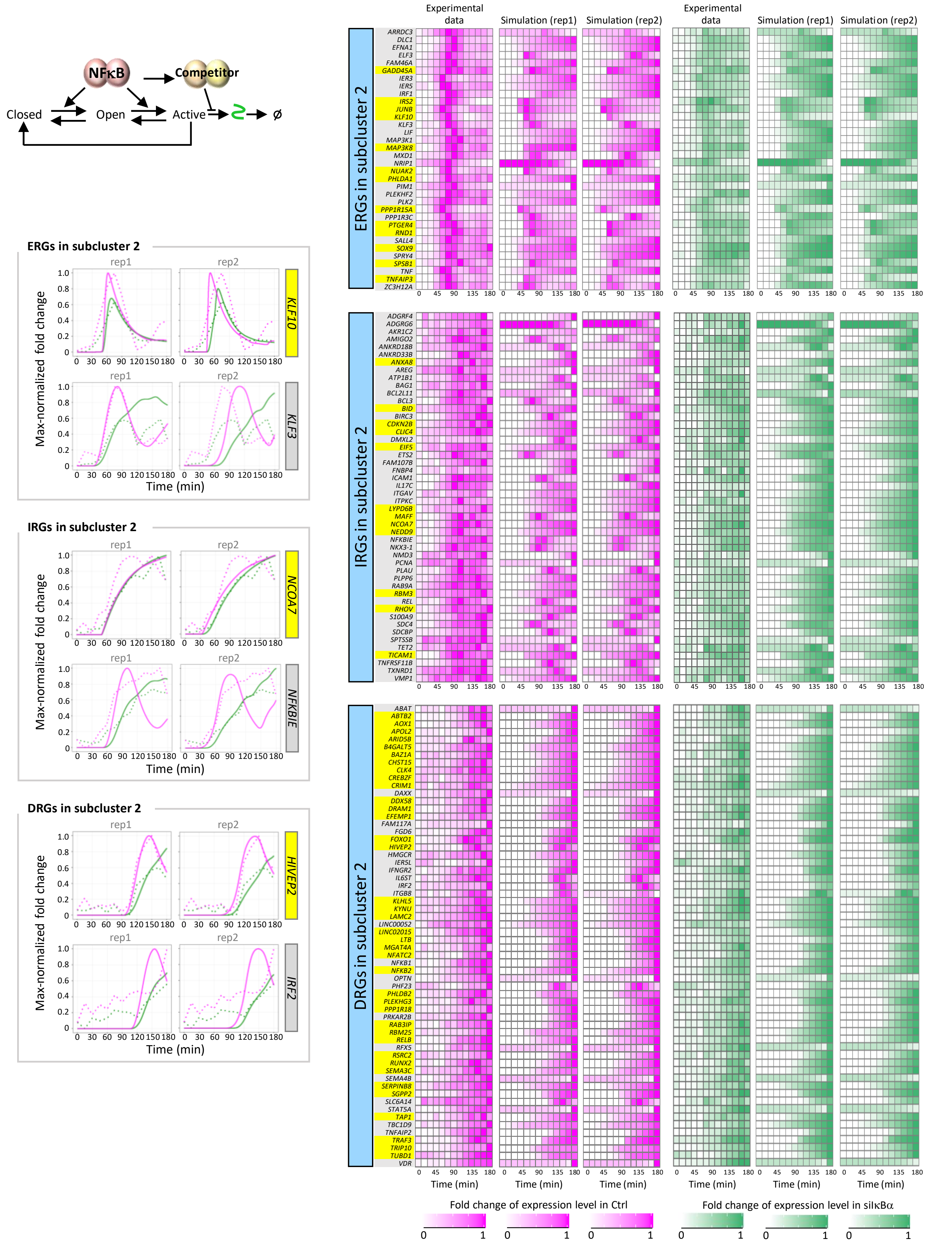

Supplementary Figure 16. Model v4 accounts for both post-induction repression and reduced expression of early response genes

Schematic diagram of the model v4. Heatmaps of the time course gene expression from experimental results and data-fit from model v4. Genes highlighted with yellow and grey indicate whether the model is acceptable or not (definition of acceptable genes: nRMSD in Ctrl > 0.5, nRMSD in siIκBα > 0.39 and AUC of fold change in expression should be Ctrl > siIκBα or max-fold induction should be Ctrl > siIκBα). Line graphs of representative genes from ERGs in subcluster 2, IRGs in subcluster 2 and DRGs in subcluster 2 showing good and bad fits between the simulation results and the experimental data.

Motif analysis results at ATAC-seq peaks in promoter regions in Ctrl (focused on κB motifs)

	ERGs in subcluster 2			IRGs in subcluster 2			DRGs in subcluster 2		
	P-value	Targets %	Motif	P-value	Targets %	Motif	P-value	Targets %	Motif
0 min									
30 min	1e-3	7.69 %	REL	1e-3	5.26 %	RELB			
75 min									
120 min	1e-4	38.10 %	RELB				1e-6	20.59 %	NFκB-p65-Rel

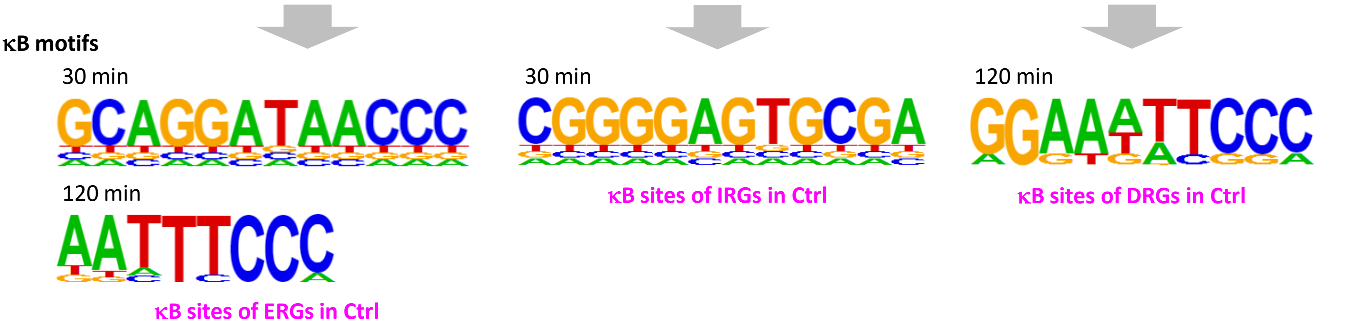

Motif analysis results at ATAC-seq peaks in promoter regions in siIκBα (focused on κB motifs)

	ERGs in subcluster 2			IRGs in subcluster 2			DRGs in subcluster 2		
	P-value	Targets %	Motif	P-value	Targets %	Motif	P-value	Targets %	Motif
0 min	1e-6	38.89 %	RELB	1e-4	13.64 %	RELB			
30 min	1e-8	35.71 %	RELB	1e-10	31.82 %	RELB			
75 min	1e-7	23.53 %	REL	1e-8	33.33 %	REL	1e-7	14.71 %	RELB
120 min	1e-7	19.23 %	NFκB-p50, p52				1e-5	41.67 %	REL

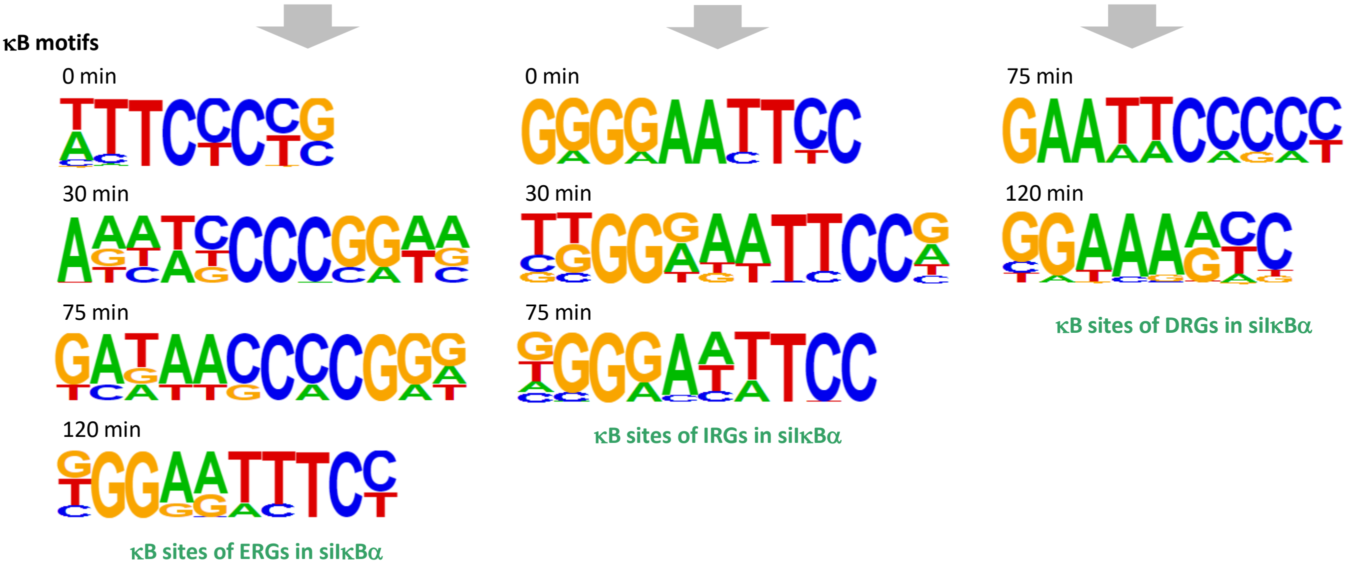

Supplementary Figure 17. κB site enriched ATAC-seq peaks in promoter regions of ERGs, IRGs and DRGs in subcluster 2
κB site enriched aggregated single-cell ATAC-seq peak regions in promoter regions of ERGs, IRGs and DRGs in subcluster 2 were identified in Ctrl (Control) and siIκBα (IκBα knockdown). For κB site enriched peak regions at each time point, κB site detected regions were extracted and merged for multiple time points.

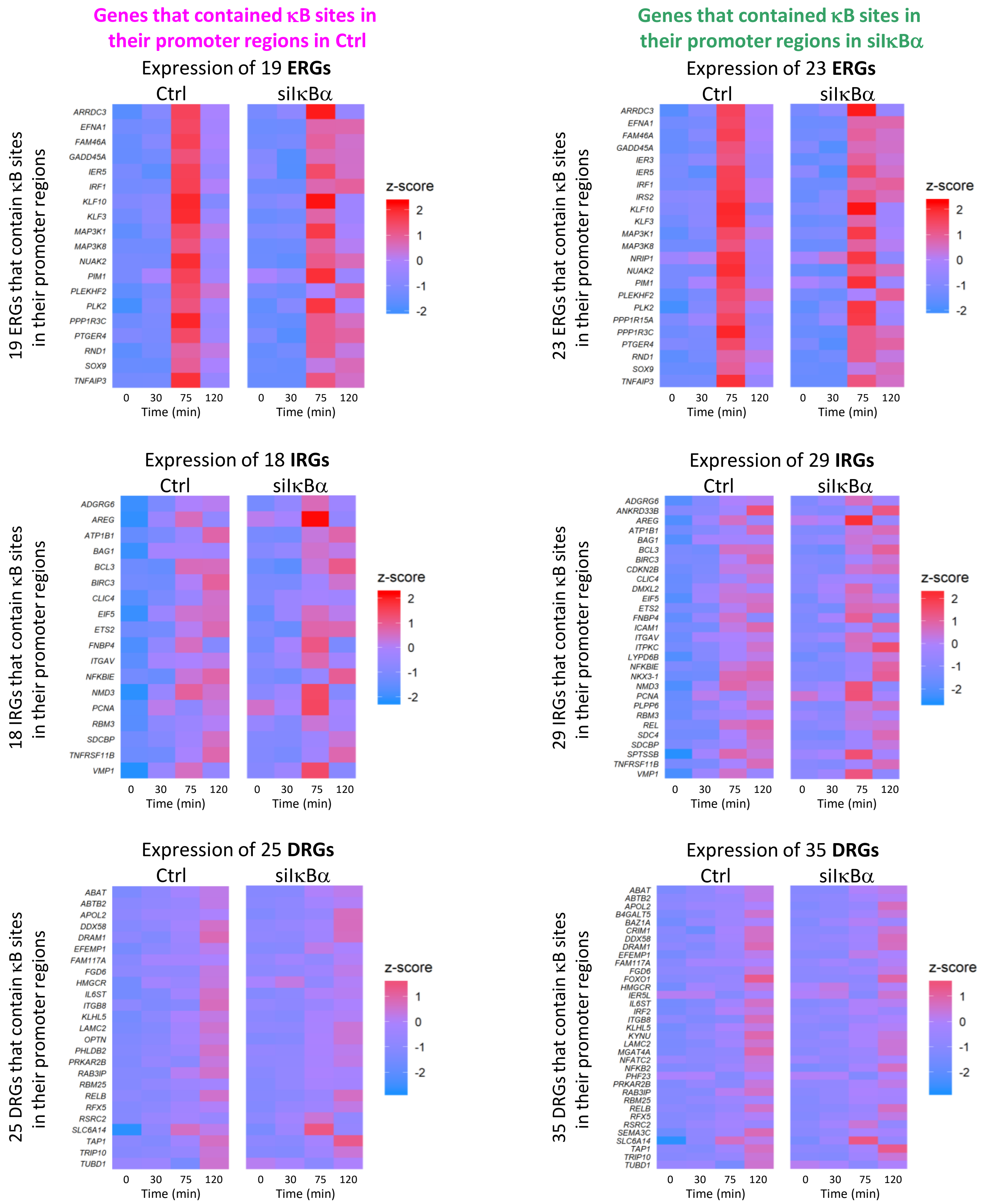

Supplementary Figure 18. Expression of ERGs, IRGs and DRGs in subcluster 2 that contained κB sites in their promoter regions

Z-score normalized time course fold change in expression of ERGs, IRGs and DRGs in subcluster 2 which contained κB site detected aggregated single-cell ATAC-seq peak regions in promoter regions (± 500 bps TSS).

		Time (min)												
		0	15	30	45	60	75	90	105	120	135	150	165	180
Ctrl (control)	rep1	529	596	733	816	824	655	784	773	628	698	682	744	708
	rep2	1447	1454	1508	1507	1550	1516	1457	1510	1427	1179	1456	1372	1404
	Total	1976	2050	2241	2323	2374	2171	2241	2283	2055	1877	2138	2116	2112
siIκBα (IκBα knockdown)	rep1	358	632	501	540	615	373	578	384	429	532	530	606	575
	rep2	1331	1438	1216	1364	1201	1370	882	989	1093	804	972	1083	953
	Total	1689	2070	1717	1904	1816	1743	1460	1373	1522	1336	1502	1689	1528

Supplementary Table 1. Number of cells used to calculate the mean nuclear NFκB abundance
Number of cells used to calculate the mean nuclear NFκB abundance from fixed-cell immunofluorescence for each replicate. The line graphs in Figure 1a and 1b represent the means of two biological replicates.

Final parameters from IFFL model

		Replicate 1					Replicate 2				
		K_{D1}	K_{D2}	k_{deg}	τ	$nRMSD$	K_{D1}	K_{D2}	k_{deg}	τ	$nRMSD$
ERGs in subcluster 2	*EFNA1*	0.0019928	25.484033	135.2559	1813.7321	0.18	0.0019922	25.469167	135.84312	1280.4528	0.36
	IRF1	0.001998	600.67811	721.36238	1992.6428	0.297	0.001998	601.58886	725.98031	2441.2035	0.382
	SPRY4	0.0016517	988.62978	0.6579738	1884.2928	0.112	0.0015937	998.61908	0.7019319	319.56499	0.21
IRGs in subcluster 2	*ANKRD33B*	0.0019986	0.0097208	0.0530492	4758.4274	0.314	0.0019905	0.0096085	0.0549308	4086.2815	0.245
DRGs in subcluster 2	*EFEMP1*	2.001E-05	380.74655	0.0292608	2299.645	0.221	2.099E-05	386.59903	0.020577	1117.8637	0.196
	IL6ST	2.098E-05	0.3026217	0.0752164	3979.6154	0.297	2.122E-05	0.3222135	0.0736342	3740.6976	0.25
	RAB3IP	2.078E-05	466.04915	0.0207453	2751.756	0.218	2.132E-05	436.91854	0.0231093	1314.3915	0.193
	RBM25	2.068E-05	204.51387	0.0063278	3416.192	0.22	0.00002	214.83903	0.0059375	2521.4175	0.199
	SEMA3C	0.00002	0.0335382	0.0065372	2847.7874	0.228	2.013E-05	0.0343019	0.0069697	401.67127	0.208
	TRIP10	0.00002	944.37166	0.0271759	3891.8677	0.204	0.00002	879.46744	0.028939	3046.9812	0.149

Supplementary Table 2. Final parameters from IFFL model which showed the best-fit with this model
Concordant parameter values from IFFL model are shown for genes which showed the best-fit with this model (red). The color corresponds to the background color in Figure 3a, bar color in Figure 3b and color of the heatmap in Figure 3c.

Final parameters from model v4

		Replicate 1								Replicate 2							
		k_1	k_2	K_{D1}	K_{D2}	K_{DTF2}	k_{deg}	τ	$nRMSD$	k_1	k_2	K_{D1}	K_{D2}	K_{DTF2}	k_{deg}	τ	$nRMSD$
ERGs in subcluster 2	*GADD45A*	6.094E-05	0.0069315	6.8326747	0.0070877	4.4249864	0.0001823	1467.2756	0.215	0.0000612	0.0069315	70.28606	0.127869	2.967172	0.000196	1366.452	0.262
	IRS2	6.223E-05	0.0069315	2.3744896	0.0033579	4.17E+00	0.000467	830.95288	0.261	0.00006	0.0069315	4.023761	6.20E-03	2.01E-02	0.000466	510.7857	0.245
	JUNB	0.00006	0.0069315	20.621912	0.0055929	8.28E+00	0.00028	978.2381	0.155	0.00006	0.0069315	68.78451	1.33E-01	7.63E+00	0.000259	892.7266	0.189
	KLF10	0.00006	0.0790494	100.18785	0.0010877	6.69E-02	0.0005692	1486.7168	0.109	0.00006	0.1106137	457.7014	2.17E-02	6.52E-02	0.000681	1334.059	0.107
	NUAK2	0.0002389	0.0069315	100.21873	0.0057123	7.50E-02	0.0007647	1292.2505	0.194	0.0001521	0.0069315	121.8737	1.16E-01	2.85E+00	0.00077	1239.956	0.222
	PHLDA1	0.00006	0.0069315	6.4919373	0.4372155	8.81E-01	2.011E-05	711.60669	0.36	0.00006	0.0069315	5.950145	2.58E-01	3.32E-03	2.01E-05	353.5466	0.321
	PPP1R15A	0.00006	0.0069315	173.81574	0.0153682	2.40E-01	0.00056	1036.2762	0.155	0.00006	0.0069315	377.4738	1.94E-01	3.40E-01	0.000578	781.0564	0.179
	PTGER4	0.00006	0.0069315	1.0215609	0.0614616	3.74E-01	0.0005881	1622.2741	0.143	0.00006	0.0069315	82.63871	2.51E+00	4.39E-02	0.000618	1326.89	0.174
	RND1	0.00006	0.0069315	41.441261	0.0134642	9.23E-01	0.0002473	1579.5795	0.205	0.0000659	0.0069315	363.7671	1.13E-01	1.31E+00	0.000291	1299.963	0.263
	SOX9	0.00006	0.0069315	65.861287	0.001	1.69E+02	8.934E-05	617.27131	0.124	0.00006	0.0070925	1.266778	5.74E-02	4.53E+00	9.19E-05	770.7953	0.094
	SPSB1	0.00006	0.0069315	2.4118963	0.009672	5.29E+00	0.0003302	1576.6162	0.154	0.00006	0.0069315	30.52954	1.57E-01	1.00E+02	0.00039	1644.518	0.192
	TNFAIP3	0.00006	0.0155537	157.17721	0.0071576	0.0457229	0.0004836	1539.755	0.191	0.00006	0.0199431	144.6095	0.046568	9.833913	0.000552	1252.137	0.234
IRGs in subcluster 2	*ANXA8*	0.00019	7.3716694	4.2236943	77.093614	0.0551235	0.00002	1591.1709	0.387	0.0001691	6.2425196	3.5417792	69.87054	0.016968	2.02E-05	1232.442	0.338
	CDKN2B	0.0023414	1.50557	3.8111639	81.742175	0.1916869	2.061E-05	1194.7186	0.249	0.0023219	1.4751693	3.9050249	92.64123	0.225978	2.03E-05	789.3653	0.217
	EIF5	0.000163	2.004599	31.099045	79.353807	0.0086066	2.968E-05	23.237469	0.319	0.0001619	1.982126	31.981384	76.62046	0.016204	3.03E-05	11.85812	0.296
	MAFF	0.0012096	10.457971	0.001	0.0363799	0.1357894	0.0007702	1268.9892	0.329	0.0012079	10.680647	0.001	0.033519	0.134688	0.00077	1141.522	0.365
	NCOA7	0.0001639	1.0751748	5.704312	74.379745	16.561011	0.0003273	1504.1668	0.201	0.0001643	1.0172962	9.0842476	74.90733	21.09458	0.000259	971.9605	0.188
	NEDD9	0.0001061	0.0072624	2.5604241	0.2602142	81.442486	2.824E-05	1176.2428	0.363	9.669E-05	0.0069315	2.827524	0.345669	101.6943	2.82E-05	624.542	0.325
	TICAM1	6.00E-05	0.0610048	61.77843	0.2197544	0.3724758	5.359E-05	1.65E+03	0.286	6E-05	0.0531109	60.776604	0.216081	0.316664	4.57E-05	1109.53	0.261
DRGs in subcluster 2	*B4GALT5*	1.09E-03	7.3607509	0.0478802	9.0896379	0.0350456	2.195E-05	1.23E+03	0.118	0.0010561	7.0276146	0.0478797	8.459485	0.046	4.91E-05	768.7869	0.117
	BAZ1A	1.23E-03	0.0446542	32.340332	69.213953	0.1397222	0.00002	1.94E+03	0.35	0.0012714	0.0460224	36.964197	79.09579	0.139128	2.01E-05	1579.49	0.323
	CLK4	0.0002434	11.437045	4.5605779	7.8578377	2.1353671	2.601E-05	1622.933	0.354	0.0002075	9.1934742	3.757759	18.60933	1.238629	2.56E-05	1316.358	0.312
	DDX58	0.0010822	9.9406505	5.613143	1.0852653	0.1516257	2.03E-05	2977.1374	0.388	0.0010947	10.0834	5.6968911	1.100757	0.084584	2.04E-05	2121.155	0.337
	HIVEP2	0.0005213	0.3507377	0.001	7.8190945	0.4517713	0.0005059	2767.1378	0.242	0.0005128	0.3489214	0.001	7.724586	0.456565	0.000506	2052.614	0.203
	KLHL5	1.95E-04	0.0823391	0.1098225	201.48414	0.0996198	0.00002	1.13E+03	0.151	0.0002352	0.0913462	0.1098225	194.1247	0.088752	2.09E-05	503.4247	0.142
	LAMC2	0.0006168	0.0827111	0.1733975	0.7103841	0.0242258	2.11E-05	1460.8962	0.188	0.0008098	0.0601576	0.1898816	0.702451	0.03365	2.07E-05	912.0301	0.164
	NFKB2	0.0001667	12.719184	17.625589	98.952696	0.0034073	0.00002	1404.3271	0.221	0.0001784	13.809966	14.485025	94.73779	0.004007	2.00E-05	915.3729	0.178
	PHLDB2	4.19E-04	7.749026	0.001	1.6133171	0.2709448	2.133E-05	1.77E+03	0.275	0.0004343	8.1903078	0.001	1.64751	0.195376	2.07E-05	873.2983	0.258
	RSRC2	0.000437	1.7147202	30.175572	77.328554	24.066167	3.852E-05	3032.4721	0.37	0.0004481	1.7669144	42.352644	70.8426	24.72987	3.94E-05	2466.404	0.344
	SERPINB8	1.68E-04	6.7764374	3.2403713	75.549507	0.0137878	2.005E-05	1.18E+03	0.36	0.0001667	6.1681615	1.3879058	100.0686	0.01181	2.13E-05	1076.485	0.31
	TUBD1	3.48E-03	3.7335313	0.0010316	2.2059175	0.0192208	0.00002	1.98E+03	0.293	0.0034813	3.7391336	0.0010316	2.208204	0.01917	2.00E-05	1204.961	0.292

Supplementary Table 3. Final parameters from model v4 which showed the best-fit with this model

Concordant parameter values from model v4 are shown for genes which showed the best-fit with this model (purple). The color corresponds to the background color in Figure 3a, bar color in Figure 3b and color of the heatmap in Figure 3c.

Final parameters from 3-state cycle model

		Replicate 1							Replicate 2						
		k_1	k_2	K_{D1}	K_{D2}	k_{deg}	τ	$nRMSD$	k_1	k_2	K_{D1}	K_{D2}	k_{deg}	τ	$nRMSD$
ERGs in subcluster 2	*ZC3H12A*	0.0001861	0.0553976	77.989738	0.0050788	0.000767	2464.7789	0.266	0.0001572	0.0526365	192.89209	0.008353	0.000746	2445.142	0.205
IRGs in subcluster 2	*ADGRG6*	0.0031646	0.5139928	11.466712	16.266969	2.02E-05	2419.9223	0.365	0.0031682	0.5147593	11.4624	15.34411	2.03E-05	1203.841	0.34
	AMIGO2	0.0001524	0.1333854	13.150849	142.27877	2.28E-05	519.47067	0.258	0.0001434	0.1135728	10.940529	144.9104	2.76E-05	0	0.245
	ATP1B1	0.0001608	0.0069315	33.020555	1.1123875	2.00E-05	3668.0615	0.362	0.0001608	0.0069315	37.028683	1.234141	2.00E-05	2802.641	0.337
	BCL2L11	0.0007732	0.8979535	134.33564	125.78044	8.02E-05	685.83924	0.327	0.0008046	0.8816924	131.9425	131.9471	9.30E-05	19.44306	0.311
	BID	0.0008307	2.4713916	76.102315	13.309057	2.00E-05	965.62935	0.244	0.0008637	2.6306537	69.37178	14.16907	2.06E-05	113.0864	0.222
	BIRC3	0.0031923	18.14327	0.5039428	11.231251	3.28E-05	3844.4018	0.32	0.0032095	18.155882	0.6117337	10.45337	3.31E-05	3649.231	0.273
	CLIC4	0.0016751	18.303986	86.839319	13.021112	2.00E-05	1146.7538	0.265	0.0016789	18.419813	87.011842	13.09168	2.01E-05	73.15877	0.237
	RBM3	0.0016222	0.6563842	38.761285	11.72142	2.04E-05	604.72118	0.379	0.0016223	0.657044	38.695847	11.73428	2.08E-05	604.8171	0.342
	RHOV	0.0003743	21.672474	4.9943927	0.2370228	2.01E-05	1269.2479	0.319	0.0003293	20.420224	4.9934259	0.227785	2.02E-05	19.87472	0.289
	TNFRSF11B	0.0001626	3.5520927	12.147243	17.463756	2.03E-05	3454.1991	0.317	0.0001519	3.5901758	12.281717	18.61284	2.05E-05	2790.586	0.27
DRGs in subcluster 2	*ABAT*	0.00006	0.0069315	164.12276	12.963582	2E-05	3610.2054	0.228	0.00006	0.0069315	152.03071	10.04087	0.00002	3238.19	0.22
	ABTB2	0.0007738	4.5831285	55.667654	136.64529	2.008E-05	4081.9701	0.192	0.0007618	4.4264599	53.35549	134.8479	0.00002	3338.225	0.153
	AOX1	0.0019903	4.4349379	126.32938	147.39908	2.001E-05	3297.5792	0.296	0.0018206	4.5589814	130.20619	154.2928	2.02E-05	2824.627	0.256
	FOXO1	0.0033165	1.1665208	0.001	2.3093744	0.0004489	4099.7876	0.178	0.0031115	1.1923161	0.001	2.19336	0.000515	2927.283	0.167
	IFNGR2	0.0057233	4.4071207	0.0350163	0.2923042	2.064E-05	2096.7466	0.163	0.0057492	3.9766743	0.0350163	0.282761	0.00002	768.1387	0.157
	IRF2	0.0058909	4.4652138	0.001	0.0029632	2.516E-05	6751.5566	0.262	0.00599	4.546791	0.001	0.002619	2.38E-05	5193.699	0.234
	LINC02015	0.0004865	25.349373	553.65482	18.604151	0.00002	4221.5398	0.313	0.0004757	25.341868	556.23942	17.92058	2.03E-05	3471.367	0.259
	MGAT4A	3.75E-04	36.224858	0.001	0.0278843	3.872E-05	3.83E+03	0.22	0.0003954	37.831198	0.001	0.027057	4.11E-05	2599.622	0.222
	NFATC2	2.77E-03	3.4778294	7.1288024	11.380224	3.345E-05	6.25E+03	0.167	0.0030197	3.4273643	7.0254002	11.10557	4.16E-05	5289.278	0.128
	NFKB1	1.50E-04	23.202772	6.7082856	13.112908	2.014E-05	3.56E+03	0.18	0.0001554	22.410053	6.4929008	12.61161	2.01E-05	2637.347	0.138
	PRKAR2B	0.0014864	16.315417	52.63418	121.70608	2.002E-05	3393.0461	0.249	0.0014227	15.689384	50.57367	113.4446	2.01E-05	2446.819	0.211
	RUNX2	0.0059303	7.323914	0.001	0.0202499	0.0001895	4299.7613	0.346	0.0059938	7.8638646	0.001	0.019385	0.000208	3006.316	0.345
	TAP1	0.0020978	20.514911	20.346956	198.83838	0.00002	3173.0727	0.308	0.0020978	20.514911	20.346956	198.8384	2E-05	2537.922	0.269
	TBC1D9	5.14E-03	63.479171	0.001	0.0652885	0.00002	5.88E+03	0.331	0.0051419	62.154344	0.001	0.060427	0.00002	4261.844	0.302
	TNFAIP2	0.0031986	4.017792	6.3664545	131.28104	0.00002	4278.187	0.221	0.0035116	4.1327565	6.5006749	153.4572	2.01E-05	3460.425	0.171
	VDR	0.0001365	16.459926	113.16407	11.984794	9.168E-05	3181.687	0.136	0.0001526	19.064916	105.97251	13.6288	8.77E-05	2223.886	0.148

Supplementary Table 4. Final parameters from 3-state cycle model which showed the best-fit with this model
Concordant parameter values from 3-state cycle model are shown for genes which showed the best-fit with this model (green). The color corresponds to the background color in Figure 3a, bar color in Figure 3b and color of the heatmap in Figure 3c.

Final parameters from simple model

		Replicate 1				Replicate 2			
		k_{deg}	K_D	τ	$nRMSD$	k_{deg}	K_D	τ	$nRMSD$
ERGs in subcluster 2	*ARRDC3*	0.0019976	0.0010007	2033.4571	0.636	0.0019976	0.0010007	2033.4571	0.628
	DLC1	0.0019996	0.0010123	2107.4196	0.377	0.0019995	0.0010138	1825.4286	0.434
	ELF3	0.0019971	0.0011459	2258.3896	0.608	0.0019971	0.0011459	2258.3896	0.637
	FAM46A	0.0019954	0.001003	1847.5465	0.527	0.0019807	0.0010022	1948.6336	0.56
	IER3	0.0019968	0.0010207	1261.5031	0.519	0.0019974	0.001021	1270.7837	0.607
	IER5	0.0019955	0.0010051	2140.3495	0.524	0.0019955	0.0010051	2140.3495	0.554
	KLF3	0.0019987	0.0010056	2483.6331	0.465	0.0019888	0.0010056	2483.6316	0.469
	LIF	0.0019976	0.0010012	2261.5851	0.526	0.0019978	0.0010001	2253.4588	0.523
	MAP3K1	0.0019912	0.0010049	2842.767	0.594	0.0019968	0.0010054	2836.9137	0.582
	MAP3K8	0.0019984	0.0010098	2174.2741	0.25	0.0019998	0.0010093	1308.2921	0.31
	MXD1	0.0019852	0.0010017	2755.5485	0.505	0.0019792	0.0010012	2471.0392	0.509
	NRIP1	0.0019943	0.0010149	2.9451805	0.723	0.0019943	0.0010149	2.9451805	0.844
	PIM1	0.0019965	0.0010318	2209.2968	0.82	0.0019965	0.0010318	2209.2968	0.792
	PLEKHF2	0.001987	0.0010102	2911.6192	0.193	0.0019839	0.0010092	1954.9937	0.18
	PLK2	0.0019987	0.0010048	1570.2208	0.523	0.001989	0.0010048	1474.6682	0.576
	PPP1R3C	0.0019935	0.0010078	2021.6578	0.495	0.0019909	0.0010081	2021.442	0.498
	SALL4	0.0019976	0.0010033	2277.82	0.452	0.0019823	0.0010045	2271.8512	0.447
	TNF	0.0019984	0.0010019	1409.74	0.401	0.0019971	0.0010021	1406.6511	0.426
IRGs in subcluster 2	*ADGRF4*	8.32E-05	0.0030517	15.946183	0.529	8.32E-05	0.0030517	15.946183	0.492
	AKR1C2	5.32E-05	0.0010143	2351.1266	0.531	5.32E-05	0.0010144	2336.4202	0.462
	ANKRD18B	2.01E-05	4.1294661	56.985201	0.363	2.10E-05	4.1255189	56.991087	0.314
	AREG	0.0001433	5.7447381	256.74882	0.812	0.0001432	5.7447239	256.74939	0.772
	BAG1	0.0001341	0.001	1158.7306	0.54	0.0001341	0.001	1158.6596	0.443
	BCL3	0.000142	373.57636	2005.9188	0.323	0.0001415	373.58237	1425.6347	0.302
	DMXL2	2.24E-05	0.0037294	2926.7702	0.503	2.25E-05	0.0039243	2895.1951	0.5
	ETS2	0.0019956	0.0010241	2312.9894	0.435	0.0019986	0.0010223	2199.8768	0.459
	FAM107B	0.0019881	0.0010027	4213.99	0.359	0.0019902	0.0010018	4217.4597	0.232
	FNBP4	2.03E-05	530.63514	47.774734	0.561	2.02E-05	530.73825	143.44834	0.531
	ICAM1	0.0001529	83.879721	3337.5404	0.262	0.0001561	83.286876	3351.6397	0.251
	IL17C	4.80E-05	0.0010118	3282.7709	0.56	4.80E-05	0.0010129	3283.57	0.512
	ITGAV	3.25E-05	0.00415	3195.3344	0.453	3.19E-05	0.0041371	3570.9342	0.453
	ITPKC	0.0019926	0.0010031	3222.8722	0.517	0.0019926	0.0010031	3222.8722	0.513
	LYPD6B	2.03E-05	542.37662	1415.5715	0.292	2.03E-05	542.37662	1415.5715	0.24
	NFKBIE	9.61E-05	991.27053	1665.7784	0.254	9.61E-05	991.27053	1665.7784	0.19
	NKX3-1	0.000192	573.13814	3116.1204	0.189	0.0001917	568.89412	2639.5343	0.173
	NMD3	5.33E-05	0.0010071	414.90887	0.691	5.33E-05	0.0010071	408.46543	0.591
	PCNA	0.0003596	0.0161133	14.442917	0.912	0.0003596	0.0161133	14.496164	0.765
	PLAU	0.0019977	0.0010084	3977.3573	0.429	0.0019966	0.0010177	3968.8383	0.349
	PLPP6	0.0001988	53.4775	3179.7928	0.42	0.000198	53.479749	2804.2671	0.401
	RAB9A	0.0003508	0.061583	2297.1668	0.672	0.0003508	0.061583	2297.1669	0.599
	REL	0.0019923	0.0010041	3354.6316	0.47	0.0019996	0.001004	3363.5525	0.427
	S100A9	4.36E-05	764.82252	3793.5322	0.322	4.37E-05	773.87811	3257.444	0.278
	SDC4	0.0001447	263.03157	2077.469	0.228	0.0001444	260.31179	1485.907	0.208
	SDCBP	2.30E-05	561.86889	3355.3292	0.297	2.29E-05	561.65152	2831.3706	0.256
	SPTSSB	0.0005791	0.0770193	62.138741	0.949	0.0005791	0.0770193	62.138741	0.821
	TET2	0.0019964	0.0115839	2869.3651	0.416	0.0019979	0.0116006	2139.685	0.416
	TXNRD1	0.0004375	0.0578571	397.06608	0.622	0.0004375	0.0578571	397.06608	0.514
	VMP1	0.0004211	0.0459664	1221.291	0.624	0.0004218	0.0459942	1206.4829	0.583

		Replicate 1				Replicate 2			
		k_{deg}	K_D	τ	$nRMSD$	k_{deg}	K_D	τ	$nRMSD$
DRGs in subcluster 2	*APOL2*	2.04E-05	135.57458	6521.3552	0.377	2.04E-05	135.42696	6512.6612	0.315
	ARID5B	0.0019982	0.0010088	5719.4353	0.21	0.0019985	0.0010072	4568.6946	0.178
	CHST15	2.17E-05	989.78581	4258.8572	0.174	2.13E-05	982.57593	3361.913	0.141
	CREBZF	2.03E-05	946.90009	3042.8177	0.289	2.02E-05	948.43574	2050.7034	0.279
	CRIM1	2.00E-05	885.22433	276.95216	0.192	2.00E-05	860.73098	0.2889717	0.178
	DAXX	2.07E-05	62.79923	3532.856	0.316	2.07E-05	62.79923	3532.856	0.255
	DRAM1	2.00E-05	693.60988	3251.2568	0.198	2.02E-05	695.86504	2295.8804	0.18
	FAM117A	2.01E-05	0.001	7199.452	0.283	2.01E-05	0.001	7195.6603	0.217
	FGD6	2.15E-05	911.61286	3699.5066	0.159	2.11E-05	920.5375	2806.996	0.148
	HMGCR	2.01E-05	0.0061386	3804.4778	0.45	2.01E-05	0.0061389	3808.3931	0.338
	IER5L	3.17E-05	32.139312	5766.5682	0.506	3.19E-05	32.265407	5694.4993	0.482
	ITGB8	2.14E-05	295.5095	3857.5748	0.24	2.14E-05	295.51249	2979.1222	0.219
	KYNU	2.01E-05	623.86368	1520.2028	0.202	2.00E-05	603.14502	566.07465	0.184
	LINC00052	5.32E-05	954.20232	100.46572	0.565	5.31E-05	950.25849	45.906522	0.527
	LTB	2.08E-05	5.0196228	5456.7167	0.295	2.10E-05	4.9614924	5440.6791	0.234
	OPTN	2.18E-05	0.00428	4570.1976	0.312	2.17E-05	0.0042017	4376.3542	0.252
	PHF23	2.03E-05	7.9239421	5702.4025	0.386	2.01E-05	7.97034	5046.8675	0.357
	PLEKHG3	2.02E-05	464.70559	2774.8249	0.273	2.01E-05	468.95481	1890.7137	0.246
	PPP1R18	2.05E-05	15.783818	3949.1561	0.328	2.03E-05	15.722889	3949.146	0.264
	RELB	2.04E-05	496.26535	3215.7759	0.241	2.02E-05	494.06903	2506.0776	0.198
	RFX5	2.07E-05	585.9953	2409.5664	0.143	2.14E-05	587.81555	1496.1996	0.136
	SEMA4B	2.01E-05	546.79608	2448.4849	0.172	2.01E-05	549.12108	1544.9533	0.151
	SGPP2	2.31E-05	110.14432	3291.3942	0.148	2.34E-05	110.05201	2355.4965	0.154
	SLC6A14	0.0002426	0.0218764	309.79534	0.647	0.0002426	0.0218689	307.20898	0.549
	STAT5A	2.00E-05	0.0137341	2332.6815	0.126	2.00E-05	0.0137341	1164.1101	0.127
	TRAF3	0.0001107	509.03697	2928.5339	0.216	0.0001103	509.85191	1818.1243	0.21

Supplementary Table 5. Final parameters from simple model which showed the best-fit with this model

Concordant parameter values from simple model are shown for genes which showed the best-fit with this model (blue). Genes that are in grey indicate that none of the 4 models were able to recapitulate their fold change in expression, and thus these parameters were used to create Figure 3c. The color blue corresponds to the background color in Figure 3a, bar color in Figure 3b and color of the heatmap in Figure 3c.
